# Urban-scale facade material mapping from street view images using vision–language models for circular construction planning

**DOI:** 10.1038/s41598-026-51028-6

**Published:** 2026-05-05

**Authors:** Deepika Raghu, Iro Armeni, Catherine De Wolf

**Affiliations:** 1 Department of Civil, Environmental and Geomatic Engineering (D-BAUG), ETH Zurich, Stefano-Franscini-Platz 5, Zürich, 8093 Switzerland; 2 Department of Civil and Environmental Engineering (CEE), Stanford University, 473 Via Ortega, Stanford, 94305 CA US

**Keywords:** Circular economy, Urban sustainability, Resource cadastre, Artificial intelligence, Large vision and language models, Computer vision, Engineering, Environmental sciences, Environmental social sciences, Geography, Geography

## Abstract

The lack of reliable building-level data remains an obstacle for advancing urban sustainability and circular economy practices. Here, we present URBAN-AI, a workflow that employs multimodal artificial intelligence to infer building material and facade characteristics from street-level imagery. We apply the workflow across six cities spanning high-income to low-income contexts (Zurich, San Francisco, Melbourne, Mumbai, Cape Town, and Rio de Janeiro), analysing and verifying outputs for 9,056 high confidence images, and achieving a mean composite module accuracy of 87.7% across all cities; to our knowledge, this represents the first cross-city evaluation of facade material classification using vision–language models. In all contexts, and especially in low-income and data-scarce settings, the workflow provides basic building information such as typology, material type, condition, and architectural style. The workflow also generates image-based indicators to inform downstream applications, including historical facade visual proxies, seismic retrofit visual proxies, energy retrofit visual proxies, urban morphology indicators, facade greening suitability visual proxies, and flood exposure visual proxies, to complement domain-specific models. We evaluate the approach in terms of accuracy, coverage, and cost efficiency, and contextualise results against conventional methods such as manual surveys. The study contributes two open resources: the Global Building Facade Dataset and the URBAN-AI workflow. These findings demonstrate the potential of AI-based workflows to support future efforts in circular construction, resource planning, and urban sustainability.

**Supplementary Information:**

The online version contains supplementary material available at 10.1038/s41598-026-51028-6.

## Introduction

The built environment, including cities, buildings, and infrastructure, provides essential systems for housing, work, and mobility, yet remains a major driver of resource depletion, waste generation, and climate change^1^. This duality presents a profound challenge in the 21 st century: supporting a global population projected to reach nearly 10 billion by 2050^2^ without exacerbating the environmental footprint of urban systems. Addressing these challenges is crucial for ensuring the future sustainability and resilience of urban environments. *Can we envision a future where our cities’ buildings symbolise sustainability rather than linear material extraction and waste generation?*

**Fig. 1:**
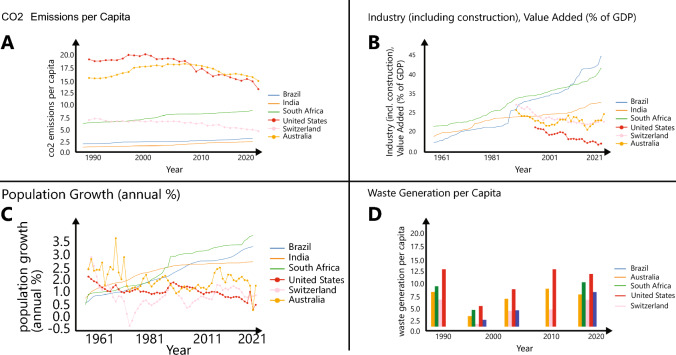
Global overview of key metrics for urban sustainability (Figure based on data from World Bank^3^). **(A) **GHG Emissions per Capita. Persistent disparities between high- and low-income countries, with particularly high per-capita emissions in countries such as the United States and Australia despite climate commitments. **(B) **Industry Value Added (% of GDP). Increasing industrial contributions in countries such as India and Brazil contrasted against a shift toward service-oriented economies in Switzerland and Australia. **(C) **Population Growth (annual %). Higher population growth rates in lower-income regions, driving demand for new housing and infrastructure, relative to slower growth in high-income contexts. **(D) **Waste Generation per Capita. Disproportionately high waste generation in high-income countries, highlighting need for improved reuse and circular strategies.

Urban development is at a critical juncture, facing the dual challenges of accommodating rapid population growth while reducing environmental impacts. World Bank indicators reveal pronounced divergences in greenhouse gas (GHG) emissions, construction-related economic activity, population growth, and waste generation across countries at different income levels (Fig. 1)^3^. High-income countries such as the United States and Australia have committed to emissions reductions under the Paris Agreement^4,5^, yet per-capita emissions remain high, around 15 metric tons, compared to approximately 2 metric tons in low to middle-income countries such as India and Brazil. Despite recent declines, these levels remain inconsistent with the 1.5–2$^\circ$C targets^5^.

At the same time, rising industrial and construction-related contributions to GDP in countries such as India and Brazil contrast with shifts toward service-oriented economies in Switzerland and Australia, reflecting different development trajectories. Combined with higher population growth rates in lower-income regions, these trends drive increased demand for housing and infrastructure, intensifying sustainability pressures. Conversely, high-income countries generate disproportionately high levels of construction and demolition waste, driven by frequent renovations and demolition-intensive redevelopment cycles enabled by relatively low material costs. Together, these patterns underscore the need for region-specific strategies that address both expanding construction demand and the management of existing building stocks within global sustainability goals. Further contextual analysis is provided in the Supplementary Introduction, Appendix A.

The circular economy (CE) promotes sustainability by redefining the traditional “end-of-life” concept, focusing on reducing, reusing, recycling, and recovering materials across the product lifecycle^6^. Although the concept has historical roots, notably articulated by Kenneth Boulding in the 1960s^7^, its application within the construction sector has expanded rapidly in recent decades^8^. This growth has occurred predominantly in high-income countries, often referred to as the Global North^9^, while material reuse practices in the Global South remain largely informal and undocumented despite long-standing traditions of resourcefulness^10^. Across both contexts, the circular shift is constrained by substantial gaps in information required for effective material reuse, with studies indicating that only about 1% of deconstructed building components are currently reused^11^.

In high-income regions, the ideal of circular retrofitting–upgrading existing buildings to improve energy efficiency and extend material lifespans^12^–is often overshadowed by the widespread practice of demolition and new construction. Similarly, in low- and middle-income regions undergoing rapid urbanisation, CE principles in construction are applied inconsistently^13^. At the same time, the built environment faces a wide range of environmental, socio-economic, and cultural challenges, including floods, heat waves, earthquakes, resource scarcity, urban density pressures, and heritage preservation^14–16^. Addressing these interconnected challenges is frequently undermined by a fundamental issue: the lack of comprehensive and reliable data on existing buildings. Because existing buildings constitute the vast majority of the built environment, improving their performance has a far greater impact on sustainability goals than focusing on new construction alone^17^. This raises a central question: *How can we leverage advancements in the digital age, particularly AI, to enhance sustainable urban environments through better data generation on existing buildings?*

**Fig. 2:**
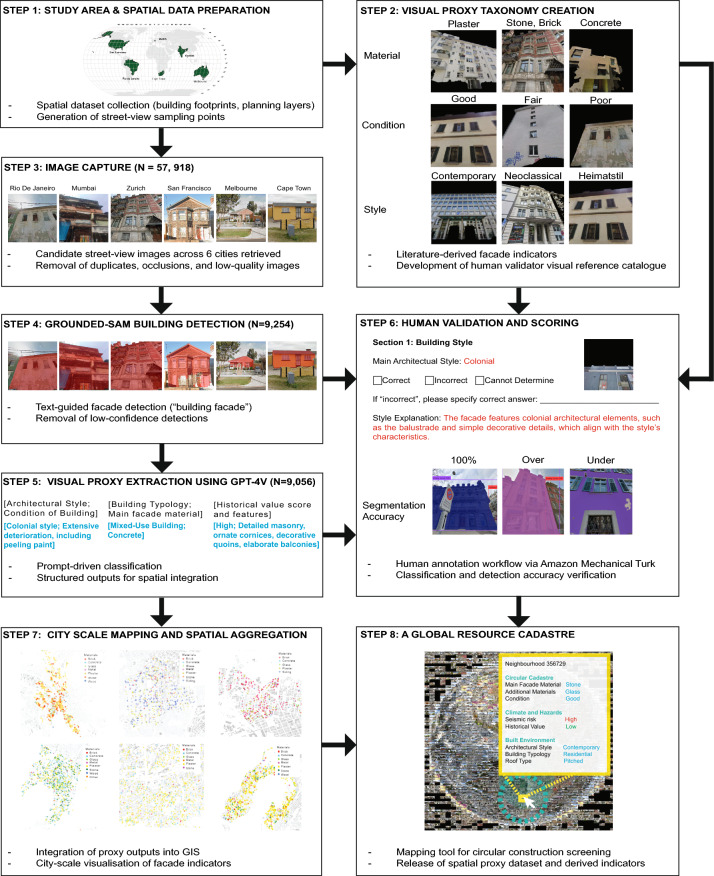
Overview of the URBAN-AI workflow. The processing and validation were conducted across all six study cities. The workflow includes study area selection and visual proxy taxonomy creation, followed by street-view image preparation and facade detection using Grounded-SAM. GPT-4V performs city-specific proxy extraction (e.g., architectural style, material, condition, and climate-related indicators), which are verified through human validation and scoring metrics. Verified outputs are aggregated through city-scale spatial mapping and integrated into the URBAN-AI global resource cadastre to support screening and planning-oriented decision-making.

Our URBAN-AI workflow employs state-of-the-art vision and language models: Grounded-SAM (a pipeline combining Grounding DINO for text-prompted object detection with the Segment Anything Model (SAM) for segmentation) and GPT-4V (Generative Pre-trained Transformer 4 with Vision, a multimodal large language model), both built on transformer architectures, to enable scalable, per-building urban analysis from street-level imagery. Street view images are sourced from public repositories such as Google Street View^18^ ensuring accessibility across data-rich and data-scarce contexts. Grounded-SAM^19^ performs text-guided facade detection and segmentation by identifying building instances based on descriptive prompts. GPT-4V^20^ then interprets the segmented facades to generate detailed descriptions of material type, architectural style, and building condition through visual reasoning.

This integration enables flexible, per-building analysis without requiring city-specific training data, contrasting with traditional approaches that rely on manual on-site surveys or rigidly structured datasets. Conventional workflows depend on labour-intensive manual labelling, where experts annotate materials (e.g., brick, concrete, wood) and components (e.g., windows, doors, beams), making them costly, time-consuming, prone to human error, and difficult to scale across heterogeneous urban environments. Existing AI datasets largely focus on street-level elements such as roads, pavements, pedestrians, sky cover, and vegetation^21^, or on interior, object-centric imagery such as furniture and appliances^22^, and therefore lack facade-level, building-instance analysis. As a result, many studies analyse limited building samples and extrapolate using proxies such as construction year, introducing uncertainty in representing the true material composition and condition of urban building stocks^23^.

URBAN-AI (Fig. 2) is a workflow for urban analysis that generates building-level datasets and spatial distributions of facade indicators–such as materials, architectural styles, and city-specific visual proxies–at a scale that is difficult to obtain without significant cost and effort. To demonstrate its applicability, six case studies were conducted across diverse socioeconomic contexts, addressing city-specific challenges such as historical facade screening in Zurich, seismic retrofit proxies in San Francisco, energy retrofit proxies in Melbourne, urban morphology indicators in Mumbai, facade greening suitability proxies in Cape Town, and flood exposure proxies in Rio de Janeiro (Supplementary Information, Appendix B). All GPT-4V outputs were manually reviewed by domain experts using a predefined verification rubric. We release both the Global-Urban-Facade-Dataset and the URBAN-AI workflow to enable reproducibility and broader adoption, particularly in regions that remain under-represented in urban sustainability research. In this way, URBAN-AI acts as a screening workflow that supports urban planning, resource management, and circular construction strategies, while complementing, rather than replacing, domain-specific models.

## Methods

The analytical scope of this study was informed by preliminary stakeholder interviews and a semi-systematic literature review (Supplementary Information, Appendix B, Sections B.1–B.2). The main research focuses on data collection, modelling, and validation procedures.

### Data sources

This study integrates spatial building data with street-level imagery to infer facade attributes at scale. Building footprint data were obtained from municipal open-data portals for Zurich, San Francisco, and Melbourne. For Mumbai, Cape Town, and Rio de Janeiro, building footprints were sourced from Google Research’s Open Buildings dataset as comparable government datasets were unavailable. For each city, a representative neighbourhood was selected, and building footprints were processed in QGIS to extract centroid coordinates for image retrieval. Detailed dataset sources, attributes, and spatial coverage are provided in Supplementary Information, Section B.3.1.

### Street-level image retrieval

Street-level imagery and metadata were accessed via the Google Street View Static API. For each building centroid, multiple candidate images were retrieved to account for varying viewpoints. Images were filtered based on (i) proximity between the camera location and the target building and (ii) camera heading, to reduce redundancy and occlusion. Images beyond a distance threshold or captured from similar viewing angles were excluded. Final image requests used default Street View parameters for field of view, pitch, and outdoor capture. Full retrieval parameters and filtering logic are reported in Supplementary Information, Section B.3.2. Additionally, a detailed breakdown of data flow and sample sizes across cities is provided in Supplementary Information, Appendix D, Table D19.

### Facade detection and masking

To isolate building facades from complex urban scenes, an open-vocabulary detection–segmentation workflow was used. The Grounded-Segment-Anything pipeline was applied using the text prompt “building facade”, in which Grounding DINO first identifies candidate facade bounding boxes and the Segment Anything Model (SAM) subsequently generates pixel-level facade masks from these detections within the same workflow. Grounding DINO provides text-conditioned object localization without pixel-level masks, while SAM performs class-agnostic segmentation without semantic labels; their combination enables precise, text-guided facade extraction. When multiple facade instances were detected, the instance with the highest confidence score and largest spatial extent was selected as the dominant facade. Images without identifiable facade detections were excluded, and confidence thresholds were applied to filter low-quality outputs. The full detection and segmentation procedure, including confidence filtering and evaluation criteria, is detailed in Supplementary Information, Section B.4.1.

### Vision–language inference of facade attributes

Masked facade images were passed to a multimodal vision–language model (GPT-4V) via its API for attribute inference. Structured prompts were used to infer facade-level attributes such as material type, architectural style, and condition. All predefined style categories used in prompts were city-specific and derived directly from the literature review summarised in Table 1. Facade materials, condition, and architectural style were inferred using constrained prompts with predefined, literature-derived category lists. Flexible prompts were used solely to generate descriptive context to aid interpretation and human verification, and were not used for categorical assignment. An explicit “other” option captured facades that could not be reliably assigned to a single predefined material class. Model outputs were parsed into structured fields for subsequent analysis. Complete prompt templates and post-processing rules are provided in Supplementary Information, Sections B.4.2–B.4.3.

### Validation protocol

Given the absence of standardized facade-level ground truth across global cities, model outputs were validated through expert review. All inferred attributes were manually reviewed by domain experts in architecture and construction using a predefined verification rubric. Validation focused on correctness of facade identification and plausibility of inferred attributes. Accuracy was computed as the proportion of correctly verified outputs at the building level. Grounded-SAM performance statistics are reported over the post-filter candidate image set (N=57,918), while GPT-4V accuracy metrics are computed on the high-confidence facade subset (N=9,254), of which N=9,056 outputs were manually validated and used for accuracy scoring. Detailed validation criteria, scoring procedures, and accuracy metrics are reported in Supplementary Information, Section B.4.4.

### Spatial analysis and indicator mapping

Verified facade attributes were aggregated to generate spatial material inventories and city-specific indicator maps. These maps represent density-normalized concentrations of image-derived facade attributes and are used to explore urban patterns relevant to material reuse, retrofitting potential, and environmental indicators. The resulting spatial outputs are interpreted as qualitative visual proxies rather than ground-truth metrics. Normalization procedures, coverage thresholds, and limitations are described in Supplementary Information, Section B.5.

## Results

### Study areas and prompt categories

A semi-systematic literature review was conducted across six cities to identify facade materials and one dominant urban challenge per city. In parallel, six semi-structured interviews were conducted with experts in manual and digital construction auditing. The review and interviews enabled a comparative assessment of traditional and digital auditing approaches, highlighting operational, economic, and technical trade-offs. In particular, they revealed the limitations of supervised, city-specific models and the potential of street-view-based approaches to generalise across heterogeneous urban contexts. This analysis establishes a workflow for city-specific prompt design by linking architectural styles, materials, and visually observable cues to dominant urban challenges (Table 1). The full literature review is provided in Supplementary Information, Appendix D.

**Table 1:** Visual Proxy Taxonomy Across the Six Global Cities, as identified through a semi-systematic literature review and used to define city-specific prompt categories for URBAN-AI (See Supplementary Information, Appendix D).

**City**	**Architectural styles**	**Urban challenges and visual cues**
ZRH	Historicism; Home Style; Art Nouveau; Neoclassicism; Classical Modernity; Contemporary	**Historical Facade Visual Proxy**: Ornate balconies and cornices, quoins, ornamentation, and decoration
SF	Mission Revival; Italianate; Queen Anne; Stick/Eastlake; Folk Victorian; Victorian Gothic; Contemporary	**Seismic Retrofit Visual Proxy**: Presence of masonry, unconfined masonry, garages, large openings, multi-story buildings
MEL	Victorian; Edwardian; Art Deco; Californian Bungalow; Classical Modernity; Contemporary	**Energy Retrofit Visual Proxy**: Large openings, roof type, HVAC, shading devices
MUM	Colonial Gothic; Art Deco; Indo-Saracenic; Modern; Contemporary; Bungalows; Informal; Utilitarian	**Urban Morphology Indicator**: Number of storeys, building typology, roof type, HVAC, utility cables
RJ	Colonial; Neoclassical; Modernist; Informal; Popular	**Flood Exposure Visual Proxy**: Large openings at lower levels, water stains, peeling paint, mold, visibility of gutters
CT	Colonial Revival; Victorian; Tudor Revival; Arts and Crafts; Modern	**Facade Greening Suitability Visual Proxy**: Number of storeys, building typology, roof type, visibility of gutters, blank wall space

### Data collection

#### Spatial data

We collected urban spatial data from open-access databases, including building footprints, building age, and city-specific attributes relevant to the identified urban challenges. Where available, temporal data supported historical analysis. Building footprints and centroid coordinates were used to retrieve corresponding street view imagery and metadata.

**Fig. 3:**
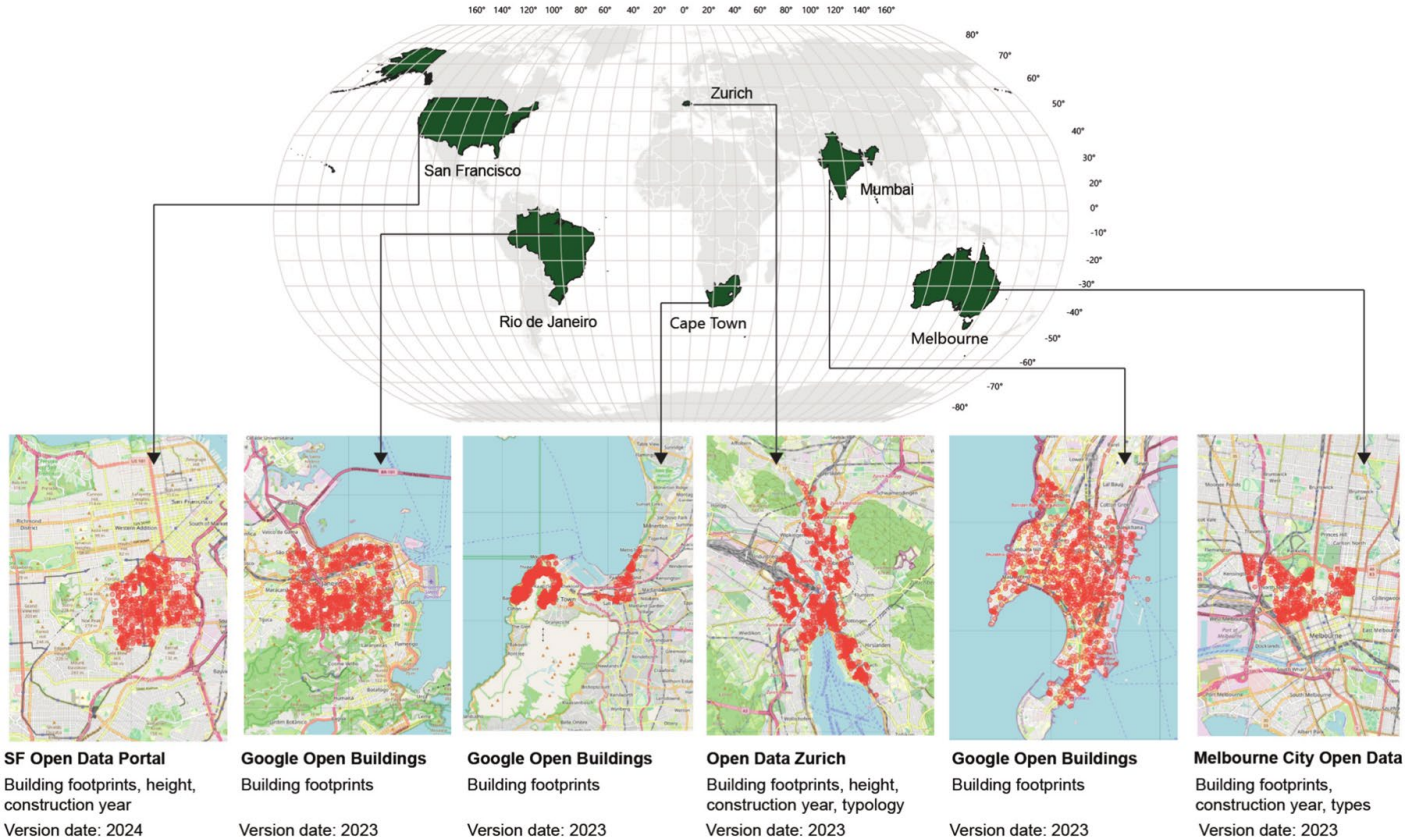
Global distribution of study zones in six cities, highlighting the specific areas analysed using street view imagery. The six study areas include San Francisco, Rio de Janeiro, Cape Town, Zurich, Mumbai, and Melbourne, each selected for its unique urban challenges and data availability. There is a notable absence of material and component information across all cities.

Across the six cities, we observed substantial variation in the quality and availability of urban datasets, alongside a consistent absence of facade material information (Fig. 3). In Zurich, detailed building identifiers are available, but heritage protection is reported only as a binary indicator, limiting assessment of historical significance. In San Francisco, construction types and permit data are available, yet the absence of material detail and building-level seismic retrofit proxies constrains quantitative risk evaluation. Melbourne links energy-use data to buildings, but lacks material composition data needed for targeted energy conservation measures. In Mumbai, Cape Town, and Rio de Janeiro, where comparable public datasets were unavailable, building footprints were sourced from Google Open Buildings, which provides geometric outlines only without additional building attributes.

#### Image data

Images and metadata for all six cities were retrieved using the Google Street View API (Table 2). Initial building counts reflected neighbourhood density and extent, with denser urban cores such as Mumbai and Zurich requiring larger samples, while San Francisco’s high-quality coverage enabled more selective sampling. Image unavailability primarily reflects Street View coverage, with higher missing rates in Zurich, Mumbai, and Cape Town, and near-complete availability in San Francisco.

For each building, centroid coordinates were computed to retrieve candidate images. Proximity filtering excluded images beyond 30 m from the target building, while a heading filter removed images with less than a 120$^{\circ }$ angular difference to reduce redundancy. Filter effects varied by urban form: irregular layouts in Rio de Janeiro and Mumbai led to higher proximity-based exclusions, while more regular street networks in San Francisco and Melbourne allowed more images to pass. Increased redundancy was observed in Melbourne’s orthogonal street grid, whereas Rio de Janeiro’s irregular, terrain-driven orientations yielded more diverse perspectives. San Francisco required minimal heading-based filtering due to its dense, multi-angle coverage.

Overall, this retrieval and filtering strategy proved robust across diverse urban morphologies, handling variability in data granularity and urban complexity. Subsequent refinement further excluded images with limited facade visibility or significant occlusions.

**Table 2:** Processing Statistics of Street View Image Dataset Across Six Cities. **Initial Selection** refers to the total number of images retrieved based on building footprints. **Unavailable** indicates images that could not be accessed due to missing data. **Exclusion** denotes the number of images removed after applying proximity and heading filters to ensure relevance and reduce redundancy. **Final Count** is the number of images remaining after processing, used for analysis. **Insights** provide contextual observations based on the characteristics of each city’s dataset.

**City**	**Initial Selection**	**Unavailable**	**Exclusion**	**Final Count**	**Insights**
ZRH	12,610	2,069	4,501	3,014	Dense urban area with some areas lacking recent updates.
SF	15,751	19	6,761	8,971	High-quality coverage allows selective focus; minimal unavailability.
MEL	11,658	40	4,549	7,069	Regular updates and well-planned urban area; good image availability.
MUM	49,324	17,543	8,909	22,872	High density and irregular urban layout lead to more exclusions.
RJ	42,583	9,205	25,313	8,065	Variable coverage with certain areas less frequently updated.
CT	32,684	1,296	23,461	7,927	Extensive sprawl and irregular building placement impact filter criteria.

### Data analysis

#### Application of vision-language model grounded-SAM for building facade detection

**Fig. 4:**
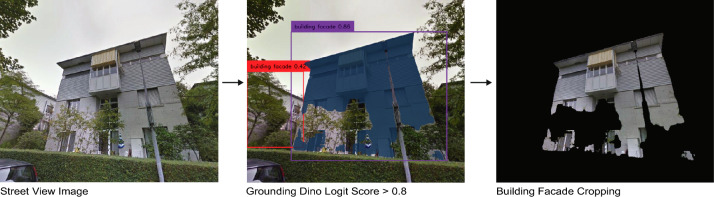
Sequence of Building Feature Extraction Using Grounded-SAM. Grounded-SAM is used in processing the raw street view image to isolate the most significant building structure for further detailed analysis. First, all potential buildings within the image are identified, followed by the application of a logit score threshold to select the most relevant structure. The selected building is then isolated, with the surrounding parts of the image masked to focus solely on the architectural feature of interest.

Grounded-SAM is used to isolate architectural features from street view imagery. Each image is first processed to detect all buildings, delineated with bounding boxes and segmentation masks (Fig. 4). Predefined criteria based on pixel area and logit scores are then applied to select the dominant building, favouring large, high-confidence segments. The selected building is isolated by masking all other image regions, enabling focused downstream analysis with GPT-4V.

Images lacking clear exterior building structures were automatically removed, as they do not support facade-level analysis. Average logit scores reflect segmentation confidence across cities, with Zurich achieving the highest mean score (0.73), indicating clear and distinct architectural features. Mumbai exhibits moderately lower scores due to complex and heterogeneous urban form, while Rio de Janeiro shows the lowest average score (0.58), reflecting challenges arising from high building density and frequent occlusions within the street-level imagery. These variations are partly attributable to data capture quality, suggesting that standardised image acquisition protocols with reduced occlusions and distortions could improve segmentation accuracy, particularly in complex urban environments.

To ensure the reliability and accuracy of the Grounded-SAM model in our analysis, we conducted a thorough evaluation of its outputs across the six cities. This evaluation involved verifying the model’s ability to correctly identify and segment buildings within the street view images. We assessed factors such as correct identification, coverage accuracy, and segmentation quality. To quantitatively determine the suitability of each image for further processing, we implemented a reward system during the verification process. The system assigns points based on criteria related to facade identification and segmentation coverage (Table 3).

To evaluate Grounded-SAM performance, outputs were manually verified across all six cities, assessing correct identification, coverage accuracy, and segmentation quality. A reward-based scoring system was applied based on facade identification and coverage criteria (Table 3). To maintain dataset quality, only images with logit scores>0.8 were retained for further processing. Approximately 15% of images were excluded due to insufficient segmentation quality or lack of identifiable building structures (Table 4), demonstrating the model’s ability to distinguish relevant architectural features from surrounding urban elements.

**Table 3:**
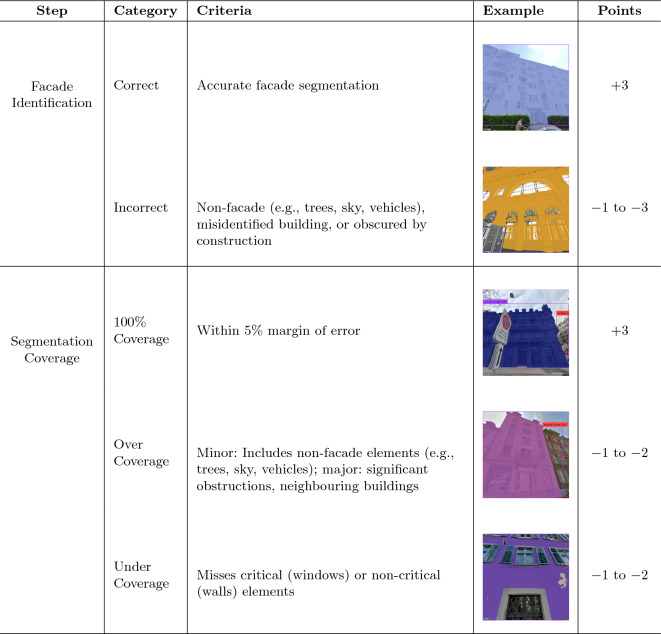
Grounded-SAM Accuracy Verification System. Each segmented facade is scored across two steps: Facade Identification and Segmentation Coverage. Correct identification and full coverage (within a 5% margin of error) each earn +3 points, while errors incur penalties ranging from −1 to −3 depending on severity. The combined score per image reflects overall segmentation quality, with higher totals indicating more accurate Grounded-SAM outputs.

**Table 4:** Grounded-SAM Analysis Across the Six Cities. Logit score distributions and accuracy verification metrics indicate consistently high facade detection performance across diverse urban contexts, with final composite scores exceeding 90% in all cities and demonstrating robust cross-city generalisation of the workflow.

**Grounded-SAM**	**ZRH**	**SF**	**MEL**	**MUM**	**RJ**	**CT**
Logit Score Analysis						
Images Analysed	3,014	8,971	7,069	22,872	8,065	7,926
Average Logit Score	0.73	0.70	0.63	0.60	0.58	0.63
Images>0.8 Score	1377	1242	1839	1753	1156	1887
Accuracy Verification						
Correct Identification	99%	89%	97%	90%	98%	99%
Incorrect Identification	1%	11%	3%	10%	2%	1%
100% Coverage	89%	98%	97%	96%	96%	98%
Over Coverage	6%	5%	1.5%	3%	3%	1%
Under Coverage	0.5%	1.5%	0.5%	6%	1%	1%
**Final Score**	**93.35%**	**95.32%**	**97.92%**	**91.32%**	**94.95%**	**98.60%**

#### Application of vision-language model GPT-4V for insights generation

A total of 9,254 images across six cities were processed using Grounded-SAM and subsequently analysed with GPT-4V, including images that were incorrectly identified or segmented in the previous step. This was done to assess GPT-4V’s robustness to upstream errors. Of the 386 incorrectly identified images, GPT-4V returned no response for 267 cases, corresponding to images lacking analysable facade content. This behaviour indicates contextual sensitivity and helps prevent error propagation from earlier pipeline stages (Table 5). The remaining 8,894 GPT-4V outputs were manually verified to assess accuracy and reliability (Table 6).

**Fig. 5:**
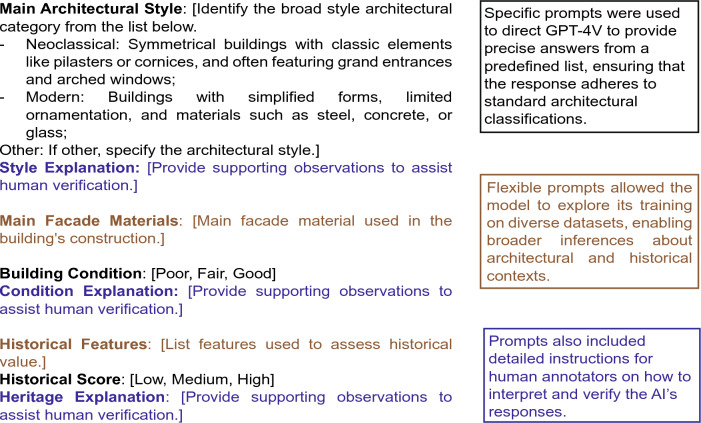
Structured Prompt Design for URBAN-AI Analysis. Prompts guide GPT-4V in analysing building and material characteristics from segmented images across cities. Prompt vocabularies are city-specific and derived from the literature-defined categories in Table 1 (see Supplementary Information for underlying literature review). **Specific prompts** constrain responses to predefined architectural categories (black), while **flexible prompts** allow broader contextual inference (brown). **Instruction prompts** support human verification (blue). The figure illustrates a schematic example rather than an exhaustive taxonomy.

GPT-4V analysis was guided by a structured prompt design (Fig. 5) that balanced specificity and flexibility. Specific prompts constrained responses to predefined architectural categories for consistency, while flexible prompts enabled contextual inference based on visual cues. For building condition assessment, Rio de Janeiro and Cape Town achieved near-perfect accuracy due to clearly visible signs of wear and weathering. In contrast, Melbourne showed lower accuracy, reflecting more subtle condition variations that are difficult to distinguish visually for both the model and human assessors.

Accuracy for city-specific attributes varied across contexts. Zurich achieved the highest performance for historical facade features due to clear and distinctive visual cues, whereas seismic and energy retrofit–relevant features in San Francisco and Melbourne were more subtle. Facade material identification was also most accurate in Zurich, reflecting the prevalence of visually distinctive materials such as stone and brick. Melbourne exhibited the lowest material accuracy due to the widespread use of modern composite materials that are difficult to differentiate at street-view resolution.

Representative examples of incorrect and uncertain GPT-4V predictions, including interpretive cases related to materials, architectural styles, condition, and climate-related facade proxies, are provided in Supplementary Information, Appendix E.

#### Overall accuracy score

URBAN-AI achieved consistent performance across cities (Table 7), with overall accuracy scores ranging from 84.66% (San Francisco) to 90.30% (Cape Town) and a combined average of 87.70%. This composite score represents the arithmetic mean of Grounded-SAM facade detection accuracy and GPT-4V attribute classification accuracy. The results indicate robust performance across diverse urban contexts with varying data quality and architectural complexity. Accuracy is influenced by expert annotation bias and the subjective nature of categories such as building condition and mixed architectural styles. Results should therefore be interpreted as facade-level screening accuracy. Future work should incorporate inter-annotator agreement and curated benchmark datasets.

**Table 5:** GPT-4V Data Retrieval and Processing Statistics Across the Six Cities. *Irrelevant* denotes images flagged by GPT-4V as lacking architectural content. *Critical* images show over- or under-coverage from Grounded-SAM. *Relevant* images capture the target facade and support accurate analysis. All outputs were manually validated.

**Insights on GPT-4V Generation**	**ZRH**	**SF**	**MEL**	**MUM**	**RJ**	**CT**
Data Retrieval						
Total Images Processed	1377	1242	1839	1753	1156	1887
Incorrect by Grounded-SAM	15	12	18	17	11	19
GPT-4V Flagged as Irrelevant	14	10	16	15	9	18
GPT-4V No Response: Critical Images	8	7	11	10	6	12
GPT-4V No Response: Relevant Images	10	8	12	11	7	14
Total Images for Manual Validation	1345	1227	1786	1732	1105	1861

**Table 6:** Human Verification Accuracy on GPT-4V Output. Classification accuracy across four attribute categories (architectural style, building condition, city-specific visual proxies, and facade material), reported per city and averaged across all six cities.

**City**	**Style**	**Condition**	**City-Specific**	**Material**	**Overall**
ZRH	71%	81%	78% (Historical facade proxies)	95%	81%
SF	65%	87%	71% (Seismic retrofit proxies)	72%	74%
MEL	72%	79%	66% (Energy retrofit proxies)	71%	72%
MUM	86%	99%	77% (Morphology indicators)	93%	89%
RJ	73%	99%	71% (Flood exposure proxies)	89%	83%
CT	72%	99%	76% (Facade greening proxies)	83%	82%
**Overall Classification Score for All Cities (%)**					**80%**

**Table 7:** Overall Accuracy Scores Across Cities. Overall Accuracy is computed as the arithmetic mean of Grounded-SAM facade detection accuracy and GPT-4V classification accuracy for each city; the combined average reflects the mean of these composite scores across all six cities.

**City**	**Grounded-SAM Accuracy (%)**	**GPT-4V Accuracy (%)**	**Overall Accuracy (%)**
ZRH	93.35%	81.00%	87.15%
SF	95.32%	74.00%	84.66%
MEL	97.92%	72.00%	84.96%
MUM	91.32%	89.00%	90.16%
RJ	94.95%	83.00%	88.97%
CT	98.60%	82.00%	90.30%
**Combined Average Overall Accuracy Score (%)**			**87.70%**

### Creating a global cadastre: City-specific mapping

**Fig. 6:**
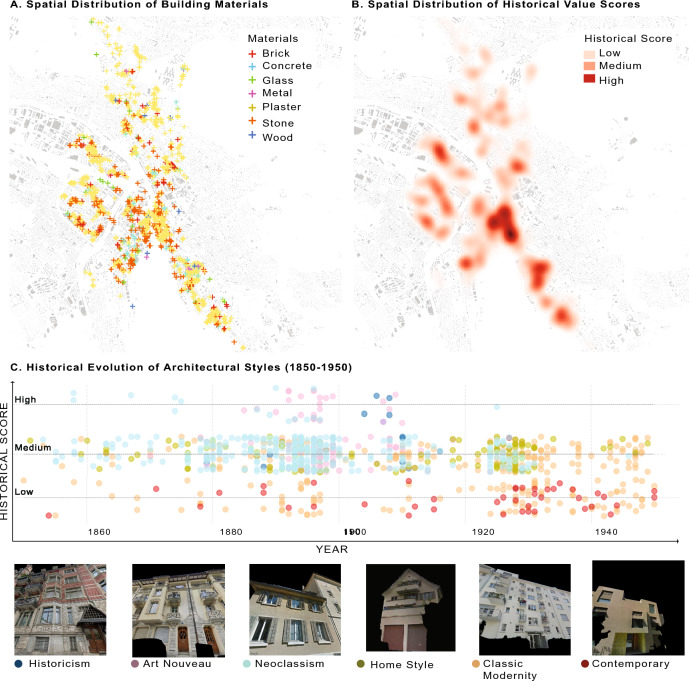
Urban Analysis in Zurich. All panels are derived from available street view imagery processed through Grounded-SAM and GPT-4V. **(A)** Spatial distribution of building facade materials across the city, including brick, concrete, stone, and plaster. **(B)** Distribution of historical facade visual proxy scores, highlighting relative spatial patterns and priority areas. **(C)** Evolution of architectural styles from 1850 to 1950, captured through GPT-4V’s analysis of architectural elements and styles, providing insights into historical development trends. Images generated using QGIS v3.42.3 (https://qgis.org) and Python v3.10 (https://www.python.org).

**Fig. 7:**
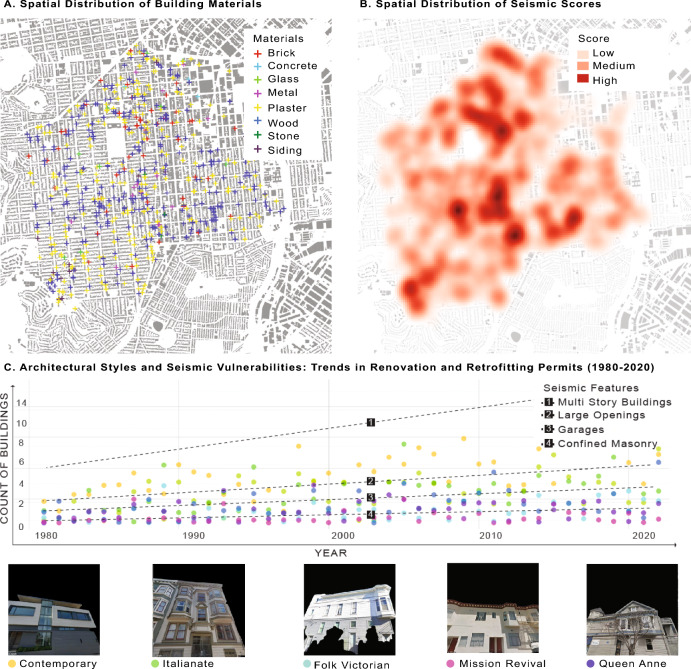
Urban Analysis in San Francisco. All panels are derived from available street view imagery processed through Grounded-SAM and GPT-4V. **(A)** Spatial distribution of building facade materials highlighting predominant materials and cladding types such as siding, brick, and plaster, especially concentrated in the northeastern and central parts of the Mission District. **(B)** Spatial distribution of seismic retrofit visual proxy scores, highlighting relative spatial patterns and priority areas. **(C)** Historical evolution of architectural styles from 1850 to 1950 alongside seismic retrofit visual proxies like large openings, confined masonry, and multi-story buildings. Images generated using QGIS v3.42.3 (https://qgis.org) and Python v3.10 (https://www.python.org/).

**Fig. 8:**
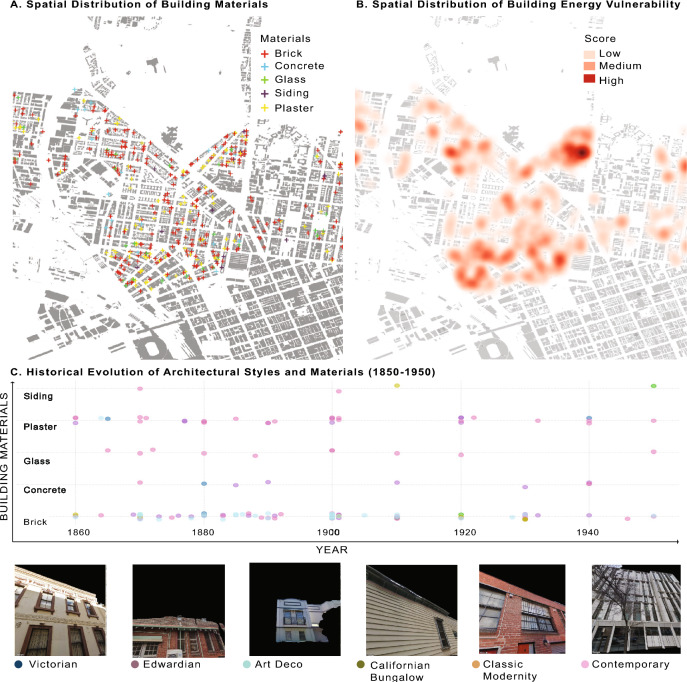
Urban Analysis in Melbourne. All panels are derived from available street view imagery processed through Grounded-SAM and GPT-4V. **(A)** Spatial distribution of building materials highlighting predominant materials such as concrete and cladding types such as siding. **(B)** Spatial distribution of building energy retrofit visual proxy scores, highlighting relative spatial patterns and priority areas. **(C)** Historical evolution of architectural styles from 1850 to 1950, showing the trends in building materials alongside the development of prevalent architectural building styles in the area. Images generated using QGIS v3.42.3 (https://qgis.org) and Python v3.10 (https://www.python.org).

**Fig. 9:**
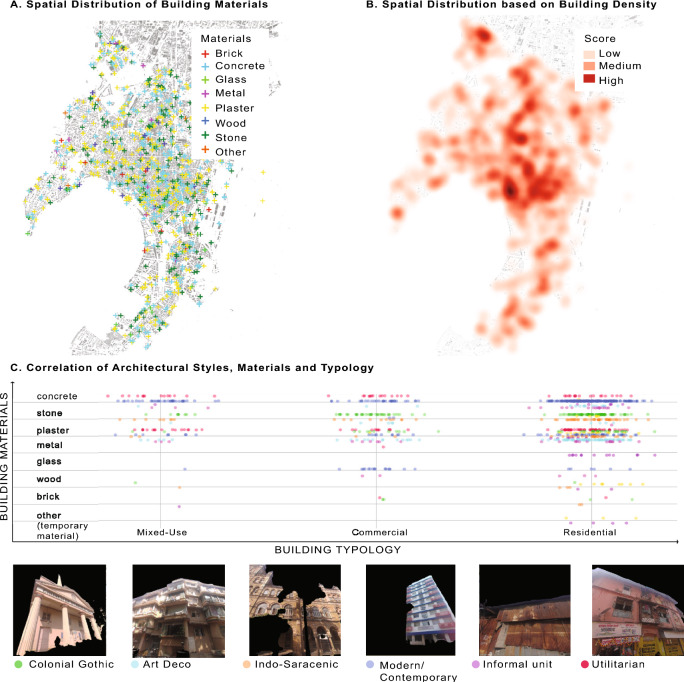
Urban Analysis in Mumbai. All panels are derived from available street view imagery processed through Grounded-SAM and GPT-4V. **(A)** Spatial distribution of building facade materials, showing the diversity of materials such as concrete, brick, and stone across the study area. **(B)** Spatial distribution based on building density indicators, highlighting relative spatial patterns and priority areas. **(C)** Correlation between architectural styles, materials, and building typologies, providing insights into the relationship between material use and building function across different urban zones, with architectural styles ranging from Colonial Gothic to informal or temporary units. Images generated using QGIS v3.42.3 (https://qgis.org) and Python v3.10 (https://www.python.org).

**Fig. 10:**
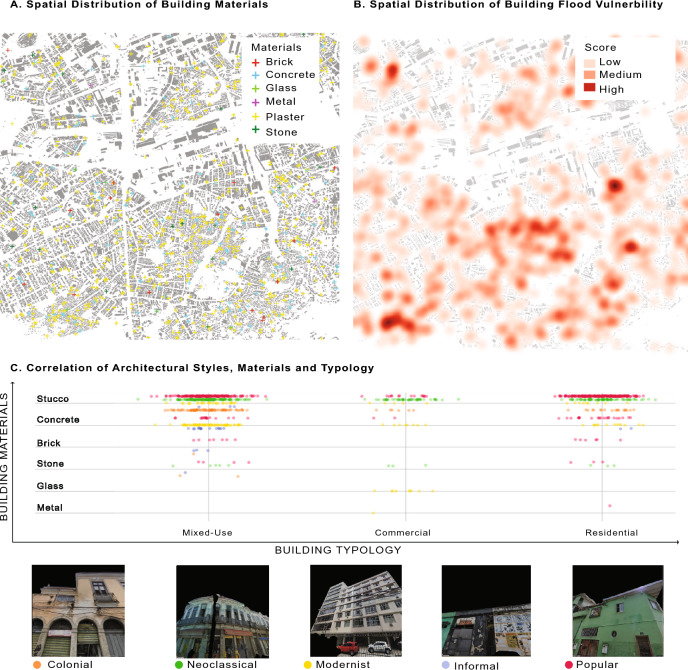
Urban Analysis in Rio de Janeiro. All panels are derived from available street view imagery processed through Grounded-SAM and GPT-4V. **(A)** Spatial distribution of building facade materials, highlighting the prevalence of concrete, brick, and plaster across the study area. **(B)** Spatial distribution of building flood exposure visual proxy scores, identifying areas of higher proxy values that coincide with densely built regions. **(C)** Correlation between architectural styles, materials, and building typologies across the study area. Images generated using QGIS v3.42.3 (https://qgis.org) and Python v3.10 (https://www.python.org/).

**Fig. 11:**
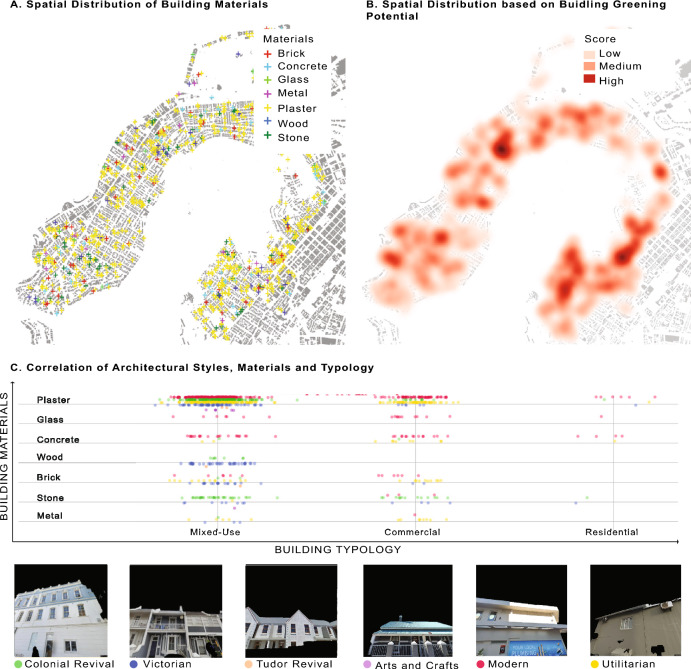
Urban Analysis in Cape Town. All panels are derived from available street view imagery processed through Grounded-SAM and GPT-4V. **(A)** Spatial distribution of building facade materials, showing a diversity of materials including plaster, brick, and concrete. **(B)** Spatial distribution of building living facade and greening suitability visual proxies, highlighting relative spatial patterns and priority areas. **(C)** Correlation between architectural styles, materials, and building typologies, showing how different materials are linked to various architectural styles ranging from older ones such as Colonial Revival and Victorian to more modern styles like Arts and Crafts and Utilitarian. Images generated using QGIS v3.42.3 (https://qgis.org) and Python v3.10 (https://www.python.org).

This section presents a comparative analysis of six cities, each associated with a dominant urban challenge. Results are organised into three panels per city (See Figs. 6,7,8,9,10,11). Panel A shows the spatial distribution of facade materials. Panel B maps facade-level visual proxies linked to a city-specific challenge (e.g., seismic retrofit, energy retrofit, or flood exposure), and should be interpreted as relative spatial patterns derived from available street view imagery. Panel C provides city-specific analysis shaped by data availability and research focus: in Zurich and San Francisco, it examines temporal evolutions in architectural styles and associated facade features; in Melbourne, it highlights prevailing architectural style compositions and transitions; and in Mumbai, Rio de Janeiro, and Cape Town, it focuses on relationships between architectural styles, materials, and building typologies.

#### Case study 1: historical facade visual proxies in Zurich

In Zurich, facade material distributions show clear spatial patterns relevant to urban conservation efforts (Fig. 6). Panel A indicates a concentration of brick and concrete facades in the historic city centre, reflecting the persistence of traditional materials in older neighbourhoods. Panel B highlights high historical facade visual proxy scores in the old town, identifying it as a priority area for conservation-oriented screening. Panel C shows a temporal shift from Historicism and Neoclassicism toward Classical Modernity and Contemporary styles over the past century. While newer styles are increasingly prevalent, historically significant facades remain spatially concentrated, supporting their prioritisation in conservation planning.

#### Case study 2: building seismic retrofit visual proxies in San Francisco

In San Francisco, facade material mapping and seismic retrofit visual proxies reveal spatial patterns relevant to retrofit prioritisation (Fig. 7). Panel A identifies siding and plaster as dominant materials and cladding types, while Panel B highlights clusters of medium to high seismic retrofit proxy scores, particularly in the Mission District. Although most buildings exhibit moderate proxy values consistent with stringent seismic regulations, some contemporary buildings show higher scores due to features such as multi-storey configurations and large ground-level openings. Panel C indicates an increase in permits for multi-storey buildings from 1980 to 2020, reflecting densification trends. Mission Revival buildings, which commonly exhibit facade features such as unreinforced masonry and large ground-level openings, show higher retrofit activity, consistent with facade features associated with increased seismic vulnerability. Buildings exhibiting garages and unreinforced masonry features also show increased permit activity, reflecting ongoing retrofit efforts targeting known seismic vulnerabilities.

#### Case study 3: building energy retrofit visual proxies in Melbourne

In Melbourne, facade material distributions and energy retrofit visual proxies also reveal patterns relevant to retrofit screening (Fig. 8). Panel A shows widespread use of materials and cladding types such as brick, plaster, and siding, while Panel B identifies zones with elevated energy retrofit proxy scores. Contemporary buildings with large openings tend to exhibit higher proxy values due to potential heat gain or loss, whereas older Victorian buildings may reduce heat transfer through smaller openings and thicker masonry walls. Panel C illustrates a historical transition from Victorian and Edwardian styles to Californian Bungalows and Classical Modernity, suggesting that older building stock may warrant targeted retrofit attention.

#### Case study 4: urban morphology indicators in Mumbai

In Mumbai, facade material mapping reflects a dense and heterogeneous urban fabric (Fig. 9). Panel A shows concentrations of concrete, plaster, and glass, particularly in central areas such as the Fort district, where formal and informal constructions coexist. Panel B highlights high urban morphology indicator values in dense central zones associated with informal settlements and high-rise residential buildings, highlighting infrastructure and planning challenges. Panel C shows that Colonial Gothic and Indo-Saracenic styles are prevalent in mixed-use and commercial buildings, while modern and utilitarian styles dominate residential areas, reflecting ongoing redevelopment pressures alongside historical layers. The presence of temporary materials such as sheet metal, tarpaulin, or composite panels is seen in informal residential extensions.

#### Case study 5: building flood exposure visual proxies in Rio de Janeiro

In Rio de Janeiro, facade material distributions and flood exposure visual proxies reveal spatial exposure patterns (Fig. 10). Panel A shows widespread use of concrete and plaster. Panel B indicates higher flood exposure proxy scores concentrated in southern areas of the study zone. Panel C links architectural styles, materials, and typologies to relative flood exposure: older Colonial and Neoclassical buildings, typically constructed from stone or brick, tend to show lower facade-level exposure proxies, while more modern structures using plaster, glass, and concrete are more prevalent in residential and mixed-use typologies and exhibit higher exposure. Multi-storey and mixed-use buildings show increased vulnerability at lower levels.

#### Case study 6: facade greening suitability visual proxies in Cape Town

In Cape Town, facade material distributions and facade greening suitability visual proxies highlight opportunities for heat mitigation strategies (Fig. 11). Panel A shows diverse material use, with concrete predominating. Panel B identifies buildings suitable for green infrastructure, particularly those with flat roofs and large blank walls that support green roofs or vertical greening. High-density areas also emerge as priority zones for greening screening due to elevated heat exposure. Panel C shows that Colonial Revival and Victorian buildings, common in mixed-use areas, are typically built with plaster and brick and have fewer blank wall surfaces and more complex roofs. In contrast, modern utilitarian buildings often use concrete and metal, with larger blank walls and flat roofs, making them more suitable for facade greening interventions.

#### Applications in circular construction

The city-scale mapping, operationalised through URBAN-AI, functions as an upstream decision-support workflow for circular construction by translating facade-level visual indicators into spatial planning inputs. The workflow supports four sequential steps: (i) facade attribute inference from street view imagery, (ii) spatial aggregation of material and condition indicators at neighbourhood scale, (iii) qualitative screening of buildings for reuse, retrofit, or recycling potential, and (iv) integration into planning or procurement decisions.

In Zurich, URBAN-AI identifies spatial clusters of buildings with brick and stone facades that overlap with historically protected zones and active renovation areas. When subsets of these buildings are scheduled for refurbishment, the facade-derived material inventory enables planners to anticipate the presence of reusable masonry prior to demolition. This facilitates early coordination with nearby restoration projects requiring material compatibility, reducing reliance on virgin materials and long-distance transport. In rapidly expanding cities such as Mumbai, the dominance of concrete and metal facades across high-density redevelopment areas enables neighbourhood-scale screening to anticipate material recovery opportunities ahead of major redevelopment phases, informing logistics and sequencing of reuse or recycling strategies. In Cape Town and Rio de Janeiro, facade material types combined with condition indicators–such as surface degradation, moisture exposure, and visible structural wear–enable prioritisation of buildings for targeted repair, retrofit, or climate adaptation interventions. In these contexts, the workflow supports early-stage screening rather than detailed engineering assessment, helping decision-makers identify priority zones for intervention under limited data availability.

### Overall metrics

The URBAN-AI workflow integrates vision–language models to enable rapid and cost-efficient urban facade analysis (Table 8). Data retrieval and preprocessing, including building selection and prompt preparation, required approximately one hour of technician time and $5 in Google Static Maps API costs for 1,000 images, resulting in a total of $35 for this phase.

Facade detection using Grounded-SAM and insight generation using GPT-4V were performed on local computing resources, requiring a one-time hardware investment of approximately $1,000. Operational costs were minimal, with estimated electricity consumption of $0.10 and GPU usage of approximately $2.40 for the full processing duration. GPT-4V API access incurred an additional $100 and enabled insight generation within approximately 70 minutes, without the need for specialised model training.

Optional quality assurance by expert reviewers required approximately one hour and $100. In total, processing 1,000 images required approximately $337.40 and just over three hours of execution time, involving one technician and, where applicable, two experts. Processing time and verification effort may increase if higher levels of detail or validation are required, depending on stakeholder needs.

**Table 8:** Summary of Time, Cost, and Personnel Requirements. Resource estimates for the URBAN-AI workflow in processing 1,000 images for urban facade inspections.

**Phase**	**Cost**	**Time**	**Personnel**
Data Retrieval	$35	1 hour	1 technician
Building ID (Grounded-SAM)	$2.40	30 min	None
Insight Gen. (GPT-4V)	$100	40 min	None
Quality Assurance	$100	1 hour	2 experts (if req.)
**Total**	$**337.40**	**3 hrs 10 min**	**1 tech + 2 experts**

## Discussion

Our approach leverages street view imagery and AI, providing a scalable and generalisable method for analysing the global built environment. This method democratises access to technology and addresses inherent data challenges thereby advancing the implementation of circular economy practices in construction. It is particularly beneficial in regions lacking extensive infrastructure or specialised local data, making it a practical workflow for both fully developed and rapidly urbanising areas. Despite its strengths, our approach faces challenges related to the temporal relevance of data, image resolution, and coverage inconsistencies. These limitations can impact the accuracy and depth of our analyses, particularly in areas with outdated or sparse street view imagery. However, the method’s scalability and generalisability offer pathways to overcome these obstacles through future integration with higher-resolution images and real-time data streams. For example, much like Ecobot, which enables real-time environmental data collection via mobile apps, URBAN-AI could be enhanced to incorporate real-time building data^24^. This would allow for live audits and assessments, making the workflow even more responsive to ongoing urban changes. Real-time data integration would strengthen its applicability in fast-changing urban environments, providing policymakers with up-to-date information for sustainability, resilience, and retrofitting efforts.

Furthermore, it is important to note that as the assessment indicators are derived from facade-level visual cues rather than physical simulations or sensor data, they should be interpreted as a qualitative screening workflow rather than quantitative measures of performance. Due to the absence of globally consistent facade-level ground truth data across cities, the evaluation relied on human verification. This introduces subjectivity, particularly for visually ambiguous or minority classes such as mixed-material facades or heavily modified buildings. Quantitative benchmarking against fixed test sets is an important direction for future work once standardized, facade-level datasets become available.

A key strength of our methodology lies in its potential to make advanced technological tools accessible for sustainable urban planning and resource management practices, especially in low and middle-income countries. The use of publicly available street view imagery and open-vocabulary models like GPT-4V enable a broad range of stakeholders to engage in analysing and optimising the use of construction materials. Such accessibility empowers communities and policymakers in resource-constrained settings to participate actively in sustainable development efforts. By lowering the barriers to entry, our approach facilitates a more inclusive and equitable contribution towards global sustainability goals. See nnn Information, Appendix F, for further discussion of limitations and interpretations.

## Conclusion

Applying URBAN-AI across six cities revealed distinct, city-specific facade patterns relevant to each local urban challenge, with composite accuracy scores ranging from 84.66% to 90.30%. In Zurich (87.15%), stone and brick dominate older districts, with historical facade features tracing a clear stylistic evolution from Historicism to Classical Modernity (1850–1950). San Francisco (84.66%) showed concentrations of siding, brick, and plaster in the Mission District, where unreinforced masonry and large ground-level openings align with ongoing seismic retrofit permit activity. Melbourne (84.96%) exhibited widespread brick, plaster, and siding, with contemporary buildings flagged for elevated energy retrofit proxy scores due to large openings. Mumbai (90.16%) presented a heterogeneous fabric of concrete, plaster, and glass, with high urban morphology indicators in dense central zones where informal and high-rise typologies coexist. Rio de Janeiro (88.97%) showed concrete and plaster dominance, with modern residential and mixed-use structures exhibiting higher flood exposure proxies relative to older Colonial and Neoclassical stone buildings. Cape Town (90.30%) demonstrated strong facade greening potential in modern utilitarian buildings with flat roofs and large blank walls, particularly in high-density, heat-exposed zones. Together, these results demonstrate that a single, prompt-configurable workflow can surface actionable, locally meaningful screening indicators across diverse socioeconomic and climatic contexts, achieving 87.7% average accuracy without city-specific model retraining.

More broadly, this work advances the integration of vision–language models with urban sustainability and circular economy practices. By harnessing street view imagery and open-vocabulary reasoning, URBAN-AI provides a scalable, generalisable workflow that democratises technology access and enhances understanding of construction materials across urban landscapes. Beyond academia, it offers a practical digital tool for policymakers, urban planners, and communities, contributing to global efforts toward sustainable development, reduced environmental impact, and enhanced resilience of urban ecosystems.

Looking ahead, continued advances in vision–language models present opportunities to refine granularity and annotation accuracy, enabling identification of individual architectural elements such as doors and windows for more detailed energy assessments. Access to temporal data could further support longitudinal analysis of building change, degradation, and policy outcomes, informing strategies for maintenance, conservation, and renewal. Most importantly, URBAN-AI is deployable today and extensible beyond the applications presented here - to gentrification, urban sprawl, infrastructure decay, and disaster response. By empowering communities in rapidly urbanising regions to engage in informed, sustainable development, this methodology contributes a practical foundation for equitable urban transformation. The datasets and code are publicly available for further research and development.

## Supplementary Information

Below is the link to the electronic supplementary material.

Supplementary Information

## Data Availability

Street-level images were accessed via Google Street View. This study releases only derived outputs, including facade-level annotations and aggregated metadata in compliance with Google Street View’s non-commercial use terms. This data is publicly available for further research and development at the URBAN-AI repository: https://raghudeepika.github.io/URBAN-AI/.

## References

[1] United Nations. *Yale Center for Ecosystems + Architecture: Building Materials and the Climate: Constructing a New Future* (Technical report, 2023).

[2] United Nations. *Department of Economic and Social Affairs, Population Division: World Population Prospects 2022: Summary of Results* (Technical report, 2022).

[3] World Bank: World Bank Open Data. https://data.worldbank.org (2015).

[4] United Nations Framework Convention on Climate Change (UNFCCC): Paris Agreement. Adopted at COP21, Paris, Bonn, Germany (2015).

[5] Black, S., Parry, I.W.H. & Zhunussova, K.: Is the Paris Agreement Working? A Stocktake of Global Climate Mitigation. Imf working paper, International Monetary Fund, Washington, DC (2023).

[6] Kirchherr, J., Reike, D. & Hekkert, M. Conceptualizing the circular economy: An analysis of 114 definitions. *Resour. Conserv. Recycl.* **127**, 221–232 (2017).

[7] Boulding, K. E. The Economics of the Coming Spaceship Earth In (ed. Jarrett, H.) (1966).

[8] Gasparri, E., Arasteh, S., Kuru, A., Stracchi, P. & Brambilla, A. Circular economy in construction: A systematic review of knowledge gaps towards a novel research framework. *Front. Built. Environ.* **9**, 1239757 (2023).

[9] Wijewickrama, M., Rameezdeen, R. & Chileshe, N. Information brokerage for circular economy in the construction industry: A systematic literature review. *J. Clean. Prod.* **313**, 127938 (2021).

[10] Muchangos, L. Mapping the circular economy concept and the global south. *Circ. Econ. Sustain.* **2**(1), 71–90 (2022).

[11] Lismont, A. & Allacker, K.: Turning the existing building stock into a resource mine: Proposal for a new method to develop building stock models. In: *IOP Conference Series: Earth and Environmental Science,* **323**, 012070 (IOP Publishing, 2019).

[12] Tingley, D. Embed circular economy thinking into building retrofit. *Commun. Eng.* **1**, 28. 10.1038/s44172-022-00027-2 (2022).

[13] Raghu, D. & De Wolf, C. India’s Informal Reuse Ecosystem Towards Circular Construction. In *Design for Rethinking Resources* 127–137 (Springer, 2023). 10.1007/978-3-031-36554-6_10.

[14] Birkmann, J. et al. Framing vulnerability, risk and societal responses: The move framework. *Nat. Hazards.* **67**, 193–211 (2013).

[15] Güneralp, B. et al. Global scenarios of urban density and its impacts on building energy use through 2050. *Proc. Natl. Acad. Sci. U. S. A.* **114**(34), 8945–8950 (2017). 28069957 10.1073/pnas.1606035114 PMC5576775

[16] Feng, Y. et al. Architectural challenges of urban heritage conservation from a sustainable development perspective. *Journal of Civil Engineering and Urban Planning* **6**(1), 50–58 (2024).

[17] Paradis, R.: Retrofitting existing buildings to improve sustainability and energy performance. *National Institute of Building Sciences* (2016)

[18] Anguelov, D. et al. Google Street View: Capturing the world at street level. *Computer* **43**(6), 32–38 (2010).

[19] Liu, S., et al.: Grounding dino: Marrying dino with grounded pre-training for open-set object detection. arXiv preprint arXiv:2303.05499 (2023).

[20] Achiam, J., et al.: GPT-4 Technical Report. arXiv (2023) 10.48550/arXiv.2303.08774 arXiv:2303.08774

[21] Hou, Y. et al. Global streetscapes—A comprehensive dataset of 10 million street-level images across 688 cities for urban science and analytics. *ISPRS J. Photogramm. Remote Sens.* **215**, 216–238 (2024).

[22] Li, W., et al.: Interiornet: Mega-scale multi-sensor photo-realistic indoor scenes dataset. arXiv preprint arXiv:1809.00716 (2018).

[23] Bradshaw, J., Jit Singh, S., Tan, S.-Y., Fishman, T. & Pott, K. GIS-based material stock analysis (MSA) of climate vulnerabilities to the tourism industry in Antigua and Barbuda. *Sustainability* **12**(19), 8090 (2020).

[24] Ecobot: Environmental Compliance Data Collection and Reporting. https://ecobot.com/. Accessed: September 25, 2024 (2024). https://ecobot.com/

