# Supplementary material for: Urban-scale facade material mapping from street view images using vision–language models for circular construction planning
Source: Sci Rep. 2026 May 5;16:20691. doi: 10.1038/s41598-026-51028-6 (PMC13334041; doi:10.1038/s41598-026-51028-6)
Supplement: Supplementary file 1 — Supplementary Information [file 41598_2026_51028_MOESM1_ESM.pdf]

Appendix A Supplementary Introduction

World Bank [1] data indicates that high-income countries are adopting sustainable practices, evidenced by stabilizing or declining per capita carbon emissions due to initiatives like the Paris Agreement [2]. For example, emissions in the United States peaked at about 20 metric tons per capita in the early 2000s and fell to around 17 metric tons by 2020. However, the current Nationally Determined Contributions (NDCs), which outline each country's efforts to reduce national emissions and adapt to climate impacts, are projected to cut global emissions by only 11%, which is insufficient to reach the 1.5°C to 2°C temperature goals [3]. Conversely, industrial expansion in countries like India and Brazil is driving increases in emissions. India's per capita emissions rose from 1 metric ton in 1990 to about 2 metric tons by 2020, while Brazil's industrial sector now makes up over 45% of its GDP. Meanwhile, nations such as Switzerland and Australia are shifting towards service-oriented economies, with industry contributions to GDP stabilizing at 20-25%. Furthermore, rapid population growth in low and middle-income countries like South Africa, often exceeding 3% annually, contrasts with slower growth in high-income nations where rates are below 1% annually. This demographic trend is increasing demand for new housing and infrastructure in low-income regions, while high-income countries focus on renovating existing structures. Additionally, high-income countries are also exhibiting higher levels of waste output per capita. This can be attributed to frequent renovations, large-scale demolitions, and insufficient recycling practices. The United States, notably, generates over 1 billion tons of construction and demolition waste annually, underscoring the need for enhanced reuse and recycling practices. These trends highlight the urgent need for region-specific strategies that promote sustainable construction and effective waste management, aligning urban development with global environmental sustainability goals.

Appendix B Supplementary Methods

This section provides extended methodological details referenced in the main manuscript (Sections 2).

B.1 Stakeholder Interviews

Our research methodology was designed to identify the extent to which the application of language and vision models on non-proprietary, open-access imagery can support managing urban resources. This exploration began with semi-structured interviews with a select group of six stakeholders from the construction and urban planning sectors. The stakeholders were selected based on their expertise in manual and digital auditing techniques. These interviews, defined by their open-ended yet targeted nature, were instrumental in gathering nuanced insights into the operational, economic, and technical dimensions of traditional building inspection techniques. Key assessment criteria included cost efficiency, time consumption, accuracy and granularity of data captured, types of data used, labour and personnel requirements, operational challenges, and the overall scope of traditional methods.

B.2 Literature Review

Our study encompassed six major cities across different continents: Zurich (Europe), San Francisco (North America), Melbourne (Australia), Mumbai (Asia), Cape Town (Africa), and Rio de Janeiro (South America). These cities were selected to represent a broad spectrum of urban environments, characterised by diverse geographic locations, architectural styles, and historical contexts. To set the scope of our study, we focused on building material inventorying across all cities and selected six distinct urban challenges tailored to the specific needs of each location, while acknowledging that some of these challenges may have broader applicability across all cities. In Zurich, we assessed the historical facade visual indicators of buildings given the city's rich historical architecture. San Francisco's building seismic retrofit visual indicators were analysed due to its vulnerability to earthquakes, while in Melbourne, we focused on building energy-related retrofit visual indicators to address sustainability in response to growing energy demands in the city. Mumbai's study centred on urban density-related visual challenges, reflecting its rapidly growing population. In Cape Town, we examined buildings for urban facade greening suitability visual indicators, important in the context of increasing temperatures and climate variability. Finally, in Rio de Janeiro, we investigated building flood exposure visual indicators due to the city's susceptibility to frequent flooding. This diverse selection of environmental challenges ensures that our findings are comprehensive and applicable across various global urban settings, highlighting the need for location-specific data to inform effective urban planning and sustainability measures. A semi-systematic literature review of existing manual and digital methods addressing these challenges in the selected cities was conducted, which helped identify the necessary information required and the gaps pertinent for our approach.

B.3 Data Collection

The next phase of our research involved the collection of a comprehensive dataset including raw spatial data as well as street view imagery and related metadata.

B.3.1 Spatial Data

In Zurich, data was sourced from Open Data Zurich, maintained by the City of Zurich. This included the building footprints, their height, construction year, typology, and heritage status. In San Francisco, building locations and structural data were sourced from the San Francisco Open Data Portal managed by the City and County of San Francisco. This dataset includes building footprints and permit data detailing various building modifications and repairs. In Melbourne, datasets provided by the City of Melbourne Open Data platform include building information such as footprints, construction dates, refurbishments, number of floors, and usage types. For Mumbai, Cape Town, and Rio de Janeiro, no government-maintained datasets providing detailed building information were available. Consequently, building footprints were sourced from Google Research's Open Buildings dataset. This collection features 1.8 billion building detection's across a substantial inference area of 58 million square kilometres, focusing on regions including Africa, South Asia, South-East Asia, Latin America,

and the Caribbean. We used QGIS, an open-source Geographic Information System (GIS) software to extract geospatial coordinates from the building footprints across all cities for a specific neighbourhood in each. Comma-separated pairs of latitude and longitude coordinates were parsed for the subsequent image and metadata retrieval.

B.3.2 Street View Data

In the subsequent phase of our study, metadata was retrieved from the Google Street View Static API, which offers details such as image availability, geographical coordinates, panorama ID, capture dates, and copyright information. A filtering process was employed to: a) exclude locations where images were unavailable via the API, b) filter buildings based on proximity, ensuring that the images captured the relevant building by considering the distance between the camera location and the target, and c) filter based on heading, referring to the camera direction in terms of angle degrees from true north, to avoid redundancy and enhance the accuracy of our urban mining analysis.

- Filtering based on proximity: We used the Pyproj library, which incorporates the Geodesic functions from the GeographicLib library, for geospatial calculations that account for the Earth's curvature and different reference systems. These calculations helped determine the distances between the locations of buildings and their corresponding street view images. A cut-off distance of 30 meters was established through empirical observations to ensure that images are closely associated with the relevant buildings, maintaining the quality of the dataset by avoiding images that might include perspective distortion or unrelated buildings.
- Filtering based on heading: To ensure diverse perspectives of each building's facade, we excluded images where the difference in camera angle (delta heading) was less than 120° from other images taken at the same location. Sometimes, the same location was used to capture images of different target buildings, leading to overlap in viewpoints. This filtering ensured that each retained image provided a distinct view of the current target building by minimizing redundancy.

The refined dataset was used to make precise image requests through the same Google Street View Static API. These requests are formatted as HTTP URLs, embedding various parameters that specify the details of the images to be fetched. The location coordinates are used as inputs, provided in a URL-encoded format. For our requests, no specific 'heading' value was added; therefore, the API automatically calculates a value that directs the camera towards the specified location from the closest photograph's point. This ensures that the captured images are optimally aligned with our objectives, focusing on accurate visual representations of urban building facades. Additionally, default parameters for the field of view, 'fov' and 'pitch' were maintained. The default fov is 90°, providing a broad horizontal field of view, while the default pitch is set at 0°, positioning the camera horizontally. This setup was seen to be optimal for capturing street-level images that are flat and wide enough to encompass the entire facade. The 'source' parameter was set to 'outdoor', limiting searches to outdoor captures and ensuring that the imagery used in our study represents the

external characteristics of buildings without the inclusion of images of indoor environments. 'Size' specifies the output size of the image in pixels. It was set to return images 600 pixels wide, and 400 pixels high.

B.4 Data Analysis

The street view image dataset serves as the base for our subsequent assessment of building facades and materials using AI.

B.4.1 Application of Vision-Language Model Grounded-SAM for Building Facade Detection

Facade detection and segmentation were performed using the Grounded-Segment-Anything framework, which integrates open-vocabulary object detection via Grounding DINO with pixel-level segmentation using the Segment Anything Model (SAM). Grounding DINO was first applied to complex street-level imagery using the text prompt "building facade" to identify candidate building instances through bounding boxes. Segmentation, in this context, refers to the generation of pixel-level masks corresponding to detected facade regions, produced by SAM using bounding box prompts from Grounding DINO. The resulting segmentation mask creates a digital overlay that outlines the identified building facade, visually separating it from surrounding urban elements such as adjacent buildings, vegetation, and vehicles. Grounding DINO outputs confidence scores (logits) for each detected bounding box, representing the likelihood that the detection corresponds to a building facade. When multiple facade instances were detected within an image, the instance with the highest logit score and largest pixel area was selected to ensure that subsequent analysis focused on the dominant building. Images without identifiable facade detections were excluded from further analysis. A higher logit score indicates greater confidence in the detection, reflecting stronger visual evidence that the detected instance corresponds to the target facade. To ensure dataset quality, a minimum logit threshold was applied to filter out low-confidence detections. Logit scores across all accepted detections were averaged to evaluate the overall reliability and consistency of the the detection. The average logit score was computed as follows:

- L_{sum} represent the sum of logit scores for all accepted detections.
- N_{det} represent the number of accepted detections.

The average logit score is then given by:

$$\text{Average Logit Score} = \frac{L_{\text{sum}}}{N_{\text{det}}}$$

Due to the zero-shot nature of the task and the absence of standardized facade-level ground truth data, segmentation quality was evaluated through visual verification. A member of the authoring team assessed the accuracy of each facade segmentation output. To systematize this evaluation, a scoring rubric was developed, as no established benchmarks were available for comparison.

- **+3 Points**: Correct Facade Identification — Segmentation accurately identifies and covers the building facade without errors. A correct facade identification is critical as it ensures that the data extracted is relevant and precise, justifying the maximum score.
- **-1 to -3 Points** (Varies by Severity): Incorrect Facade Identification — This category includes errors such as segmenting non-facade elements, facades under construction, or interior images. Minor issues, like a facade under construction, result in a smaller deduction since they still technically identify the correct structure, albeit in an incomplete state. However, major errors, such as misidentifying non-facade elements as the main building, lead to a more significant penalty due to the greater impact on the analysis.
- **+3 Points**: 100% Coverage — This score is awarded when the segmentation achieves near-perfect coverage, within a 5% margin of error for slight over or under-coverage. This high score reflects the importance of complete and accurate coverage in ensuring that the building facade is fully captured for analysis.
- **-1 to -2 Points** (Minor to Major): Over-coverage — Occurs when unnecessary elements, such as trees or vehicles, are included in the segmentation mask. The severity of the penalty is determined by how much these additional elements obstruct or interfere with the main facade. Minor issues result in a smaller deduction, while significant over-coverage that obscures the facade leads to a greater loss of points.
- **-1 to -2 Points** (Non-Critical to Critical): Under-coverage — This penalty is applied when parts of the facade are missed, which can significantly impact the accuracy depending on the importance of the missed elements. Non-critical areas might result in a minor deduction, whereas missing critical structural features of the building facade would incur a larger penalty.

The final segmentation accuracy score is computed by summing all individual segmentation scores and dividing this sum by the maximum possible score of 6 for each image. Importantly, scores cannot be negative, since deductions are capped and no further assessments are conducted for incorrect facade identifications. This result is then converted into a percentage:

- S_{sum} denote the sum of all segmentation scores.
- S_{max} denote the maximum possible score.

The segmentation accuracy is therefore calculated as:

$$\text{Segmentation Accuracy} = \left(\frac{S_{\text{sum}}}{S_{\text{max}}}\right) \times 100\%$$

Finally, the main facade was isolated from the image, with all other parts of the image masked in black, to focus the subsequent analysis on the selected structure.

B.4.2 Application of Vision-Language Model GPT-4V for Insights Generation

In our study, we combine the strengths of visual and textual data processing using GPT-4V to achieve a more comprehensive analysis of urban environments. To facilitate the analysis, the processed images from the previous steps are first encoded into a

base64 format, a process that converts the visual data into a string format. This encoding, along with a structured prompt is sent to GPT-4V through its API for each image. The prompt is carefully crafted to instruct GPT-4V to focus on identifying and analysing specific architectural elements and materials depicted in the segmented images.

The responses generated by GPT-4V are then processed to extract valuable data points about the building materials and conditions, as determined by the input prompt. This extraction is handled by functions that parse through the model's output, identifying and isolating key pieces of information as per the predefined fields in our dataset. The structured extraction simplifies the integration of model outputs and ensures consistency for further analyses. The prompts are specifically tailored to each city and its unique environmental or urban challenges. Additionally, all responses generated by GPT-4V undergo human verification through Amazon Mechanical Turk (MTurk), a crowdsourcing platform through which tasks can be outsourced to a distributed workforce. Experts from the field of architecture and construction were tasked with ensuring the data reliability and trustworthiness of our analytical process by agreeing or disagreeing the AI output. The accuracy is then calculated as the percentage of correctly identified elements verified by the MTurk workers.

- N_{correct} represent the number of correctly identified elements.
- N_{total} represent the total number of elements evaluated.

$$\text{GPT-4V Accuracy} = \left(\frac{N_{\text{correct}}}{N_{\text{total}}} \right) \times 100\%$$

B.4.3 Prompt Engineering

In URBAN-AI, a methodical approach to prompt engineering was adopted to optimise the efficacy of GPT-4V in analysing building and material characteristics from segmented images. The prompts were carefully crafted to be both detailed and concise, ensuring they effectively leverage GPT-4V's open vocabulary capabilities without introducing superfluous details. Each prompt was tailored to the specific urban and architectural context of the city under study, ensuring that the model's responses were highly relevant and adapted to the unique characteristics of each location. The prompts were designed to strike a balance between specificity and flexibility:

- Specific prompts were used to direct GPT-4V to provide precise answers from a predefined list, ensuring that the response adheres closely to standard architectural classifications. For instance, in Zurich:
  - Prompt Example: "Identify the architectural style of the building in this image from Zurich's old town, focusing on key elements like window type and facade material. Use the list of styles such as 'Neoclassical', 'Bauhaus', or 'Modernist'."
  - Purpose: This prompt directs GPT-4V to provide a specific answer by narrowing down the architectural style based on visible features. It constrains the AI to select from a predefined list, ensuring that the response adheres closely to standard architectural classifications relevant to Zurich's architectural typologies.

In cases where a facade did not clearly correspond to any of the predefined architectural categories derived from the literature, an "Other" option was explicitly included in the constrained response list. This category captured visually ambiguous facades, hybrid styles, heavily modified buildings, or cases where image quality or occlusion prevented confident classification. These instances were retained in the dataset and flagged during human verification rather than forced into a predefined class.

- Flexible prompts allowed the model to explore its training on diverse datasets, enabling broader inferences about architectural and historical contexts. For example:
  - Prompt Example: "Describe the architectural characteristics and potential historical significance of the building shown in the image from Zurich. Consider elements like material usage, construction era, and any visible modifications."
  - Purpose: This more open-ended prompt allows GPT-4V to leverage its wide-ranging training data, enabling it to infer and discuss broader historical and architectural contexts. It gives the model the flexibility to provide a comprehensive analysis that might include insights beyond just style identification, such as historical context or speculative insights about modifications and their purposes.

Importantly, constrained prompts were used for all categorical attributes (material, condition, architectural style), while flexible prompts were employed only for descriptive explanation and verification support; they were not used to generate material or style labels.

Prompts also included detailed instructions for human annotators on how to interpret and verify GPT-4V's responses. This was crucial in bridging the gap between AI-generated data and expert human analysis. For instance:

- Prompt Example: "Human annotators will verify the main architectural style so provide information of what you see to verify the main architectural style."
- Purpose: This compels GPT-4V to not only identify the architectural style but also to detail observable features that substantiate its classification, such as window shapes, materials used on facades, and other distinctive characteristics.

This structured approach employing both specific and flexible prompts enhances the utility of GPT4V's outputs. It ensures that the data generated is both accurate and informative, suitable for diverse applications in urban planning.

B.4.4 Human Evaluation

Human validation was conducted by domain experts with training in architecture, urban design, and construction practice. Reviewers were selected to reflect familiarity with the regional contexts represented in the study and included experts with professional or academic experience in Europe, North America, Australia, South Asia, Africa, and South America. This geographic diversity was intended to reduce region-specific interpretation bias when validating facade attributes across cities. These domain experts performed the validation tasks via a custom Human Intelligence Task (HIT) interface deployed on Amazon Mechanical Turk (MTurk), which was used as a task

distribution and data collection platform. All reviewers followed the same predefined verification rubric described below.

To support efficient and consistent verification, a structured HIT interface was developed consisting of an image container displaying the street-level building image and a form container presenting the AI-generated descriptions for review. An instructional section guided reviewers on task execution, interface use, and evaluation criteria. All reviewers were additionally provided with a standardized visual reference catalogue containing annotated examples of architectural styles, facade materials, and condition categories to support consistent interpretation across cities.

Reviewers were asked to agree or disagree with each AI-generated assertion. In cases of disagreement, reviewers were required to provide a corrected description, enabling targeted human oversight of model outputs. The interface dynamically adapted to the content generated by GPT-4V, ensuring that validation was specific to each inferred attribute.

Crowd-specific HTML components were used to integrate the interface with MTurk's backend. To ensure consistency across reviewers, all experts were provided with identical task instructions, reference examples, and validation criteria.

B.5 Overall Accuracy Score

To provide a comprehensive measure of URBAN-AI's performance on building facade imagery, an overall accuracy score is calculated as a weighted average of the individual metrics assessed by the vision-language models. The weights assigned to each metric are determined based on their relative importance in the context of building identification and insights generation.

The building-level accuracy score is computed using the following formula:

$$\begin{aligned}\text{Overall Accuracy Score} = & \; w_1 \times \text{Grounding-SAM Accuracy} \\ & + w_2 \times \text{Average Logit Score} \\ & + w_3 \times \text{GPT-4V Accuracy}\end{aligned}$$

where $w_1, w_2, w_3, w_4, w_5, w_6$ are the weights assigned to each metric, ensuring the sum of all weights equals 1. Based on our analysis, the weights were assigned as follows:

- **Grounding-SAM Accuracy:** $w_1 = 0.2$ - Accurate segmentation is crucial for delineating buildings from urban clutter, forming the basis for further analysis.
- **Average Logit Score:** $w_3 = 0.1$ - Reflects model confidence in its predictions, important but secondary to the actual segmentation and identification accuracy.
- **GPT-4V Accuracy:** $w_4 = 0.2$ - Essential for the AI's ability to correctly identify and classify building characteristics, directly impacting the quality of the dataset.

These weighted averages provide separate, comprehensive metrics that summarize the performance of the vision and language models at both the building and material levels, reflecting their combined effectiveness in urban sustainability analysis. This score helps in comparing the effectiveness of AI-driven methodologies against traditional methods and in identifying areas for further enhancement.

B.6 Analytical Framework

To create a comprehensive framework that addresses both broad urban circular economy initiatives and city-specific challenges, we designed our study around two sets of key questions tailored to the unique environmental and urban contexts of six major cities. This analytical approach, derived from our literature review, helped set the scope of our study; however, many other questions could be answered from the data collected. The first question focuses on general urban circularity insights applicable across all cities: "How can material inventory maps be created to visualise the distribution and availability of building materials in different urban areas?" This is addressed by visualising building-level material data in spatial maps, offering insights for identifying opportunities for material reuse and guiding strategic urban planning efforts toward sustainability. The second set of questions is tailored to the specific challenges faced by each city. For Zurich, we asked,"Which architectural styles should be prioritised for future conservation efforts based on their historical significance?" and "Which areas in Zurich exhibit the highest historical value and therefore warrent prioritised conservation attention?" In San Francisco, the focus was on seismic resilience, with questions like "Which architectural styles should be prioritised for future retrofitting efforts based on their seismic retrofit visual proxy indicators?" and "Which areas in San Francisco have the highest seismic retrofit visual proxy scores and require prioritised retrofitting efforts?" Melbourne's analysis centred on energy efficiency, asking, "How can we identify areas with buildings having energy retrofit visual indicators?" and "How have architectural styles and materials evolved over time?" For Mumbai, Rio de Janeiro, and Cape Town, we focused on questions such as "How does building typology and material correlate with architectural styles?" and more specifically, "Which zones exhibit indicators of high building density?" in Mumbai, "Which architectural styles should be prioritised for future retrofitting efforts based on their flood exposure visual proxies?" in Rio de Janeiro, and "Which buildings are most suitable for urban facade greening with vertical gardens or green roofs to help mitigate severe heat?" in Cape Town. These questions are addressed using facade-derived visual indicators, which function as qualitative screening proxies rather than quantitative measures of performance.

Appendix C Supplementary Results

C.1 Stakeholder Perspectives

The semi-structured interviews conducted with specialists aimed to uncover the operational, economic, and technical nuances of traditional and digital audit methods for building facades. For traditional audits, questions focused on practical aspects such as the typical costs, time requirements, personnel involved, and the level of detail achievable in the resultant data. Specialists were asked to describe the workflow, from initial site visits to the final reporting, and how these factors influence decision-making in regulatory compliance and demolition planning. Conversely, the inquiry into digital audit techniques revolved around their cost efficiency, technological requirements,

operational speed, and the scalability of digital methods in urban environments. Questions sought to determine the impact of technology like AI and Light Detection and Ranging (LiDAR) on the frequency and accuracy of building assessments.

The responses from specialists highlight the distinct contrasts between traditional and digital methods. Traditional audits were detailed by five specialists with a geographic split between the Global North and South. In the Global North, the cost ranges from $5,000 to $15,000 depending on project complexity, building components used, and the homogeneity of all facades of the structure. Specifically, an expert highlighted that for a four-story building, it would take about four to eight hours on-site and three weeks to produce the final report. These audits typically involve teams of two, including experts in chemical and waste management. One specialist shared, “The granularity is good enough for decisions related to selective demolition,” emphasising that while the information meets requirements for compliance with local regulations, detailed data on materials is typically limited to those classified as hazardous.

In contrast, the Global South features an informal reuse ecosystem that operates with significantly lower costs and different operational dynamics. Informal waste pickers play a crucial role in this ecosystem, often working under challenging conditions with minimal tools and resources. These waste pickers are typically engaged in the collection, segregation, and resale of reusable building materials from demolition sites or urban waste. Their methods are highly manual and labour-intensive, relying on personal initiative and entrepreneurial skills rather than sophisticated technology. An expert described this scenario: “The income of demolition experts or waste pickers varies according to the region in which they are located.” These workers often endure long hours, typically collecting about 60–90 kg of waste per day over 8–10 hours, with daily earnings ranging between Rs. 500 to Rs. 1500 [4].

Digital building audit techniques, as detailed by three specialists, provide substantial cost reductions—approximately 7× cheaper than traditional methods—and significant time savings by eliminating travel. One expert emphasised the efficiency of automated inspections: “With automated inspection, the results are instant, allowing for multiple inspections throughout the year,” which notably increases the frequency and effectiveness of building facade assessments. Typically, only one or two quality assurance experts are needed when AI is used. Without the use of AI, when images are manually labelled manually to identify building defects, around 10 digital surveyors are needed. A city of 100k properties would have a team of multiple full time inspectors checking a sample of properties every year. These experts use data collected digitally, including street view imagery, aerial imagery and LiDAR, with resolutions ranging from 2 to 25 cm/pixel, to meet the specific needs of diverse sectors like housing associations and insurance companies. The targeted maximum deviation from ground truth is 15%, reflecting acceptable torerance levels for qualitative screening applications, while balancing operational efficiency and data fidelity. The quality assurers oversee and verify the integrity of the automated data to ensure that digital audits meet the standards of precision and accuracy.

This juxtaposition illustrates a clear trade-off: while traditional methods provide accurate data critical for specific applications, digital methods offer broader, scalable benefits that are indispensable for frequent, large-scale building facade assessments.

In the Global South, informal methods, though less costly and less structured, often rely on local networks to identify where to find reusable materials—a process that is time-consuming, infrequent, and inefficient. One local authority respondent mentioned, “There is a dire need to initiate policy action for their social and economic upliftment,” also pointing out the stark contrast in working conditions. Furthermore, it is important to note that digital building audits, while efficient in terms of data capture and assessing the condition of buildings, do not yet focus extensively on specific building materials. Thus, it is evident that the implementation of a global resource cadastre could significantly enhance these efforts, providing a unified platform that bridges the data gaps between diverse auditing practices. Once the initial data is collected, further on-site assessments can be conducted to identify hazardous materials or verify specific details in selected buildings. The goal is not to replace manual on-site audits but to complement them with a rapid assessment database that provides an initial understanding of the current building status. This approach enables more strategic resource management and planning. While it may offer less detail in some aspects, it provides more in others, such as scalability and insights at the individual building level. The method is also tailored to the unique contexts of both the Global North and South, supporting global sustainability goals.

Appendix D Literature Review

D.1 State-of-the-Art

The global effort to implement circular construction and retrofitting of buildings faces significant hurdles due to data inadequacies. Government databases, essential for cataloguing building types, construction methods, and material usage, often lack detail, particularly in high-income nations, and are nearly non-existent in many low and middle-income ones. This deficiency primarily stems from reliance on outdated manual auditing methods, which are labour-intensive, costly, and unable to keep pace with rapid changes in construction practices and urban development [5]. As a result, the collection, updating, and analysis of construction data is limited, posing a significant barrier to sustainability efforts. Without accurate, comprehensive, and accessible data, strategies for effective circular practices remain limited to small-scale, niche projects, which stymie broader progress toward sustainable, resilient urban environments [6, 7]. Material intensity studies help understand the composition and reuse potential of urban infrastructure and buildings. Early research, paved the way by quantifying the stock of minerals in Zurich's urban fabric using historical data [8]. Similar efforts projected Japan's future construction material demand and potential construction and demolition waste reuse [9], while other research generates insights into the material stocks for residential buildings and transportation networks within the European Union, leveraging data on floor sizes and building age [10]. However, these studies primarily rely on statistical datasets, focusing on specific material fractions, such as minerals and metals, without granular localisation at the individual building level. This limitation highlights the need for geo-spatial explicit data for every single building instance. Geographical Information Systems (GIS) data can provide such specificity. Studies using GIS, have demonstrated the strengths of applying

geospatial datasets in identifying parts of the building stock, though without delving into the material details [11]. Existing research has leveraged GIS by analysing different building structures to localise various building types [12]. Yet, the challenge of accurately determining material composition still stands, often resulting in uncertainty due to varied data sources and assumptions based on construction periods. Recent research explores material-informed Building Information Modelling (BIM) based on on-site investigations and laser scans to assess material intensities more accurately, though this method's scalability remains in question [5]. The diversity of building types, especially non-residential ones, poses significant challenges in quantifying material stocks and flows [13]. The lack of benchmarks suitable for validating these methods adds another layer of complexity, especially due to the regional variations in construction designs and the heterogeneity of buildings' physical structures. Despite these challenges, there is a clear opportunity to improve bottom-up models' accuracy by integrating advanced remote sensing technologies and AI-enhanced methodologies. For instance, recent research has incorporated photogrammetry and image processing techniques into urban building energy models, enabling the use of actual physical attributes rather than relying on broad assumptions across large areas [14]. Computer vision enables machines to interpret visual data, but its use in identifying construction materials has been relatively restricted. Some studies have successfully identified materials such as plastics, metals, wood, and concrete within waste destined for disposal or recycling facilities [15, 16]. More recent research by [17] has focused on developing data-centric models to recognise construction materials from building photographs, prioritising the creation of high-quality datasets rather than analysing urban material stocks. This limitation hinders the broader adoption of sustainable practices in circular construction and efforts to mitigate environmental impact. Latest research that examines leveraging street view imagery and transformer models, tackles the problem of building facade material detection as a multi-label classification task [18]. However, this approach still requires extensive manual labelling of materials, which limits its scalability across regions and for different applications. This paper builds on these foundations, proposing a methodology that refines building and material data collection through AI for informing sustainable urban planning and circular economy practices through a scalable, adaptable approach.

D.2 Study Area Review

The case-study cities were selected based on their diverse environmental, socio-economic, and architectural contexts, offering a wide range of urban challenges such as historical facade indicators, seismic retrofit potential indicators, energy retrofit potential indicators, urban morphology indicators, facade greening suitability indicators, and flood exposure indicators. By choosing the cities of Zurich, San Francisco, Melbourne, Mumbai, Cape Town, and Rio de Janeiro, the study captures global variations in urban sustainability issues, providing a test-bed for the URBAN-AI workflow. Notably, building material information was not available in all cities. For a visual representation, refer to our online catalogue.

D.2.1 Challenge 1: Historical Facade Screening of Buildings in Zurich

The review of existing literature highlights a significant transition from traditional manual methodologies to advanced digital techniques in heritage assessment [1]. This shift reflects a broader trend towards integrating technological innovations for enhanced conservation effectiveness. Manual methods, including direct observation and archival research, have been foundational in historical assessments. However, these approaches face significant challenges regarding scalability and subjectivity, which can compromise their effectiveness across extensive areas. Such methods are noted for being resource-intensive and susceptible to human error, which may affect the reliability of the data collected [19, 20]. On the other hand, digital methods like photogrammetry, LiDAR, and Historical Building Information Modelling (HBIM) enhance accuracy and data integration. Despite their advantages, these digital approaches require specialised skills and are often cost-prohibitive [21, 22]. GIS and image-based analysis, including street view imagery, emerge as promising alternatives for conducting large-scale, cost-efficient urban historical assessments. Recent studies using these tools demonstrated their utility in assessing urban aesthetics and historical characteristics [23, 24]. Nevertheless, such methods predominantly capture broad urban features—such as trees, sidewalks, and facades—rather than the intricate architectural details crucial for precise historical conservation. Despite advancements in methodological approaches, detailed research on Zurich's architectural heritage remains sparse. A notable study documents architectural styles and trends in Zurich from the late 19th to the mid-20th century, highlighting prominent styles such as Historismus and Heimatstil [25]. Historismus, or Historicism, is known for its eclectic approach, combining elements from various historical periods to create visually imposing structures. Heimatstil, or Homeland Style, marks a departure towards more localised and functional architectural expressions, emphasising simplicity and the use of local materials. This movement is closely associated with the Gartenstadt, or Garden City movement, which integrates green spaces into urban planning to improve living conditions by blending nature and urban structures. Despite the rich documentation of these stylistic evolution's [25], the existing literature often overlooks individual building-level assessments, which are essential for detailed heritage evaluations. This gap highlights the need for more focused studies that can support historical value screening across large areas. Street view images offer a viable tool for detecting historical facade visual proxies relevant to such screening (Table D1). The layout, materials, and general styles of buildings are discernible, providing valuable context for heritage evaluations. Features visible from these images, such as rooflines, window styles, and facade materials, can be used to infer architectural styles and relative historical character, thus enriching the understanding of Zurich's architectural evolution (Table D2). The selection of architectural features such as rectangular windows, ornate and modern balconies, quoins, and various roof forms (Table D3) are therefore treated as qualitative visual proxies for historical significance, supporting further expert-led evaluation.

[1] In this discussion, "historical" refers to the intrinsic value, age, and past significance of buildings, while "heritage" relates to their recognized or designated cultural importance and preservation efforts.

Table D1 Summary of Required Features for Building Historical Significance Assessment.

Feature	Detection Street View	Relevance
Geometry [19, 26]	Partially	Essential for structural assessment and restoration planning; 2D perspective may limit depth analysis.
Location [27]	No	Provides context and spatial analysis crucial for urban management; can be obtained from metadata.
Colour [26]	Yes	Indicative of material condition and environmental effects.
Material [19, 28]	Partially	Crucial for conservation decisions; detectable for exposed materials.
Construction Techniques [27]	Partially	Offers insights into historical building practices; only visible elements can be analyzed.
Condition [19, 26]	Partially	Critical for maintenance prioritization and conservation strategy development; external signs of wear can be detected.
Historical Context (eg. construction period) [28]	No	Cannot be directly detected but inferred from building age.
Architectural Style [28]	Partially	Constructive elements (e.g., wall, columns, pillars) provide insights into the period and cultural context of the building; detailed accuracy may be limited due to the variety and overlap in styles or modifications over time.
Facade Components [29]	Yes	Cornices, pilasters, balconies, quoins, decoration.

Table D2 Assessment of Zurich Architectural Styles [25] from Street View Images

Style	Description (from street view)
Historicism (Historismus)	Large pilasters (Kolossalpilasters) and elaborate window treatments are visible. The eclectic mix is noted, though rich details may not be fully appreciable from street level.
Home Style (Heimatstil - Reformarchitektur)	Traditional craftsmanship and use of local materials are identifiable. Simplicity and integration with nature are suggested through visible materials and design.
Art Nouveau (Jugendstil)	Organic forms and flowing lines, such as ornamental gates and curved windows, may be visible. The expressiveness might not be fully appreciated from a limited view.
English Cottage Style (Englischer Landhausstil)	Varied rooflines, asymmetrical facades, and natural materials are visible, suggesting an informal layout and flexibility in floor planning.
Neoclassicism (Neoklassizismus)	Orderly architectural elements like aligned windows and symmetrical doorways can be identified, though the full grandeur and scale may be underappreciated.
Classical Modernity (Klassische Moderne)	Clean lines and functional design, such as flat roofs and horizontal windows, reflect the style's emphasis on practicality.

Table D3 Key Historical Facade Visual Proxies[29, 30] from Street View Images

Feature	Sub-Feature	Visual Cues	Significance
Windows	Rectangular Windows	Standard vertical or horizontal windows, typically with a simple frame.	Common in both historic and modern buildings, showcasing versatility across eras.
	Bay Windows	Projection outward from the main walls of the building.	Found in more ornate historical buildings, enhancing light and views.
Balconies	Ornate Balconies	Intricate designs and detailed railings.	Reflect Zurich's rich artistic past, common in Baroque or Victorian eras.
	Modern Balconies	Simplified, minimalistic railings.	Indicative of 20th-century modernist trends, focusing on functionality.
Ornamentation	Ornate Cornices	Decorative moulding along the top edge of the building primarily for aesthetic purposes.	Intricate mouldings with motifs, typical in historical contexts.
	Modern Cornices	Moulding along the top edge of the building for functional purposes.	Simplified, clean lines focused on functionality in modern architecture.
	Quoins	Accent blocks at the corner of walls.	Indicative of the building's historic structural and aesthetic considerations.

D.2.2 Challenge 2: Building Seismic Retrofit Visual Proxies in San Francisco

Traditional seismic assessments, which often require on-site inspections by teams of structural engineers and researchers, use standardised protocols like the Agibilità e Danno nell'Emergenza Sismica (AeDES) form in Italy [31] or Federal Emergency Management Agency (FEMA) forms in the United States [32]. While these methods provide in-depth insights into a building's seismic resilience, they are resource-intensive and not scalable for large-scale evaluations across urban areas due to the extensive time and manpower they require [33]. Recent advancements have seen a shift towards using street view images combined with computer vision technologies to enhance the efficiency of seismic assessments. Particularly, the application of deep learning models, such as Convolutional Neural Networks (CNNs), has been pivotal. These models are trained on large datasets of labelled images to identify and classify building features that serve as visual proxies for seismic risk potential, such as the presence of soft-story buildings—structures known for their susceptibility to earthquake damage due to open ground floors [34, 35]. San Francisco is recognised for its high seismic risk, with studies indicating a 98% probability of a damaging earthquake of magnitude 6 or

greater occurring within the next 30 years [36]. Economic and environmental impacts are significant concerns, with estimated annual losses from building-related earthquake damage in the United States totalling approximately $6.1 billion, over 61% of which is attributed to California [37]. However, challenges persist in collecting comprehensive data on building materials and inventory, necessary for large-scale seismic assessments and effective city-wide risk mitigation planning. These efforts are essential in preparing San Francisco for future seismic events, ensuring that the city can withstand and recover from these natural disasters with minimal damage. San Francisco features an array of architectural styles that reflect its cultural and historical evolution [38]. From the ornate details of Victorian Gothic to the sleek lines of Contemporary buildings, the city's landscape is a testament to its dynamic past and ongoing architectural dialogue. Within this broad spectrum, the Mission District was chosen for seismic risk potential visual proxy assessment, given the prevalence of older building structures, which may require targeted seismic retrofitting. Street view imagery serves as an effective means for identifying key features associated with building seismic risk potential visual proxies (Table D4). The detection of soft-story configurations, and the type of building materials, along with the height and condition of buildings, offers qualitative insights for seismic assessments. Additionally, these images allow for the identification of architectural styles, enhancing our knowledge of San Francisco's architectural development (Table D5). For a detailed analysis of the visual cues observed for seismic risk potential, refer to Table D6.

D.2.3 Challenge 3: Building Energy Retrofit Screening in Melbourne

Traditional assessments of building energy efficiency typically rely on manual audits and inspections, including thermal imaging and energy consumption analysis [42], alongside engineering calculations and simulation-based benchmarking [43]. However, these methodologies require detailed information about building dynamics and all building components, a level of detail often not feasible for large areas. It also involves complex models and software that are costly and labour-intensive, making them less practical for widespread assessments. Recently, there has been a significant shift towards using street view imagery combined with computer vision to support energy retrofit screening. Techniques leveraging semantic segmentation have proven instrumental in identifying building facade materials, which serve as energy-related visual proxies for thermal properties relevant to insulation performance and overheating potential[44]. The extraction of the Window-to-Wall Ratio (WWR) from these images has also emerged as a critical parameter, significantly influencing both natural lighting and solar heat gain [45]. Although not directly measurable from imagery alone, the inferred building orientation relative to the sun's path, derived from combined street view and location metadata, enhances understanding of solar gain and shading patterns, which can support qualitative inference of solar exposure and shading conditions[46]. However, it is important to note that while some existing studies use street view imagery to assess building characteristics, they typically examine these features in isolation and not in a comprehensive manner. The City of Melbourne has embarked on an ambitious environmental strategy, setting a target to achieve net

Table D4 Key Features for Building Seismic Retrofit Screening

Feature	Detection from Street View	Relevance
Presence of Masonry Buildings [33]	Partially	Masonry buildings are commonly associated with higher seismic vulnerability in prior studies and are therefore used as a seismic risk visual proxy.
Masonry Construction Type [33]	Partially	Construction type influences structural behaviour; confined masonry is generally associated with better seismic performance than unconfined masonry and is treated as a comparative visual proxy.
Presence of Soft-Story Buildings [34, 35]	Yes	Soft-story configurations are widely recognised visual proxies for elevated seismic risk potential and are therefore important for retrofit prioritisation.
Building Typology (e.g. Residential, Commercial, Industrial) [39]	Partially	Different uses and configurations are associated with differing seismic risk profiles and inform qualitative screening of retrofit needs.
Number of Floors [39]	Yes	The height of a building can influence its seismic response, with taller buildings potentially having higher risk.
Building Material [33, 40, 41]	Partially	Different materials exhibit different seismic behaviours; material identification supports qualitative retrofit prioritisation.
Condition [33, 35]	Partially	Visible signs of deterioration are used as qualitative visual proxies that may indicate reduced structural robustness.
Location [33–35, 39–41]	No	Provides spatial context for urban-scale analysis and is obtained from metadata.

zero emissions by 2040 [47]. This initiative calls for significant transformation within the urban fabric, particularly focusing on the retrofitting of buildings to substantially reduce carbon emissions. In response to these challenges, a focused study area, characterised by a diverse mix of residential and commercial properties in North Melbourne has been selected. Melbourne's architectural narrative features a broad range of styles, from Victorian and Edwardian to Contemporary and High-Tech, each exhibiting facade characteristics that influence energy retrofit visual proxies [48, 49]. Not all buildings have been individually studied, leading to a gap in data for effective energy assessments. Street view imagery can also be instrumental for identifying features relevant to energy vulnerability screening (Table D7). The analysis of shading devices, openings in the facade and materials supports qualitative identification of buildings

Table D5 Assessment of Architectural Styles [38] from Street View Images

Style	Year	Description (from street view)
Mission Revival	1890-1915	Smooth stucco walls, red tile roofs, curved parapets, reflecting early California missions.
Italianate	1865-1885	Features decorative hoods over windows, bracketed cornices, and tall, narrow windows.
Queen Anne	1885-1910	Noted for complex rooflines, asymmetrical facades, and textured walls.
Stick/Eastlake	1880-1900	Characterized by distinctive stickwork on facades and elaborate wooden decorations.
Folk Victorian	1870-1906	Less ornate, with symmetrical profiles and front-facing gables.
Victorian Gothic	1880-1899	Elaborate and exaggerated Gothic features, common in larger public buildings.
Contemporary	1990-present	Innovative materials, unconventional volumes, avant-garde and use of novel technological advancements.

Table D6 Seismic Retrofit Visual Proxies from Street View Images

Feature	Visual Cues	Significance
Masonry Buildings	Visible brick or stone walls, often without reinforcement.	Associated with higher seismic vulnerability.
Absence of Masonry	Modern materials like steel or reinforced concrete instead of masonry.	Associated with comparatively lower seismic vulnerability.
Confined Masonry	Columns at intervals, horizontal ties at the top and bottom of walls.	Improved seismic performance relative to unconfined masonry.
Unconfined Masonry	Lack of structural confinements, continuous masonry without breaks.	Indicative of higher seismic risk potential.
Soft-Story Building	Large openings on lower floors, lack of wall support below upper floors.	Widely recognised visual proxy for elevated seismic risk potential.
Absence of Soft-Story	Uniform wall distribution, minimal large openings on lower floors.	Safer when absent, reduced seismic risk potential.

likely to benefit from retrofit interventions (Table D8), while also enabling the cataloguing of architectural styles that contextualise these energy-related indicators (Table D9).

D.2.4 Challenge 4: Urban Morphology in Mumbai

Urban morphology, which studies the form and structure of urban spaces, is crucial for understanding the spatial and cultural fabric of cities. This branch of urban studies looks at how the distribution and characteristics of buildings influence urban landscapes [52, 53]. Methods for assessing urban morphology and density often rely on tools like GIS and remote sensing data, which use metrics such as building height, floor area ratio, and land coverage ratio to gauge how developed an area is [54, 55]. While these methods provide valuable macro-level insights, they often miss the finer,

Table D7 Key Features for Building Energy Efficiency Screening

Feature	Detection from Street View	Relevance
Facade Material [44]	Yes	Different materials have varying thermal properties affecting energy efficiency.
Window-to-Wall Ratio [45]	Yes	Indicative of daylight access and potential heat gain or loss.
Orientation [46]	No	Affects solar heat gain and natural lighting; can be estimated from the building's positioning relative to the sun's path.
Shading [46]	Partially	Presence of trees, other buildings, or architectural overhangs can impact solar exposure and cooling demands.
Roof Type [50]	Yes	Influences solar potential and thermal efficiency, e.g., flat roofs may support solar panels better than sloped roofs.
Condition [51]	Partially	Visible signs of wear may indicate poor insulation properties or air leaks, affecting energy efficiency.
Age [51]	No	Older buildings may lack modern insulation but this requires historical data, not visual assessment.
Architectural Style [51]	Partially	Some styles may have inherent features affecting energy performance, such as large glass facades in modern buildings.

human-scale details that can affect how densely populated areas are experienced at street level. Street-level imagery can complement these approaches by enabling facade-derived urban morphology visual proxies. For instance, analysing facades and visible features in street view images, algorithms can classify buildings based on their observable type and usage, such as residential, commercial, or industrial. This classification helps in understanding the layout and functional zones within urban spaces [56]. A significant study by [57] used street view images to classify types of points of interest within buildings, extracting text from storefronts and categorising them into various types such as bookstores and pharmacies. This methodology leverages a vision model for storefront detection and a multi-modal approach that combines visual and textual data for precise classification. However, a major challenge in implementing this technique is the extensive manual labelling required to train the models, a time-consuming and labour-intensive process crucial for achieving high accuracy but posing scalability challenges, especially when adapting to new geographical areas or varied urban settings. In the Global South, where cities experience rapid and often unregulated growth, urban morphology screening is particularly valuable due to persistent data gaps. However, there is a notable gap in detailed morphological studies that focus on the granular aspects of building types, heights, and architectural styles in these regions, which are critical for comprehensive urban analysis [52]. Mumbai, with its contrasting sprawling slums and towering high-rises, presents unique challenges in

Table D8 Energy-Related Visual Proxies from Street View Images

Feature	Visual Cues	Significance
Presence of Large Windows	Large, expansive glass surfaces.	Can increase natural light but may lead to higher heat loss or gain, impacting energy efficiency.
Flat Roof	Horizontal rooflines, absence of pitched or sloped roofing.	Can be used for solar panel installations, contributing to energy generation and efficiency.
Presence of HVAC Units	Visible external units, often on rooftops or building sides.	Indicates active climate control, which impacts energy consumption.
Facade Shading Devices	Awnings, louvers, or overhangs.	Helps reduce solar heat gain, improving energy efficiency by reducing cooling loads.

Table D9 Assessment of Architectural Styles [48, 49] in Melbourne from Street View Images

Style	Year	Key Characteristics (from street view)
Victorian	1845-1900	Ornamental brickwork, cast iron lacework, terracotta tiled roofs, decorative skirting.
Edwardian	1901-1910	Prominent front gables, steep roofs made from terracotta tiles or painted corrugated iron, L-shaped verandas with timber embellishments and fretwork.
Art Deco	1920-1930	Curved facades, decorative brickwork, parquetry floors, cream brick veneer walls.
Californian Bungalow	1930-1940	Brick or timber construction, thick columns holding up the front veranda, single storey, low, pitched or gabled roofs.
Contemporary	2000-present	Innovative materials and designs with large windows. Minimal use of decorative elements.

data collection. Traditional methods have frequently failed to capture the extreme disparities in living conditions and building types, often leaving substantial gaps in urban data. Leveraging street view imagery helps bridge these gaps by providing consistent, facade-level observations of building height, typology, and external condition, enabling a more nuanced understanding of Mumbai's urban fabric. For instance, Art Deco Mumbai [58] has inventoried several buildings in Mumbai on an interactive map but is limited to this architectural style, possibly due to challenges of traditional data collection. In Mumbai, particularly in the Fort area, the rich tapestry of architectural styles further complicates urban density assessments. This historically significant district features Art Deco—a style noted for its streamlined modernity and local cultural motifs, Gothic Revival, Indo-Saracenic, and modern contemporary architectures [59]. These styles manifest through varied architectural elements, each adding to the district's historical narrative (Table D10). Understanding such architectural diversity in relation to spatial and cultural dimensions in Mumbai is necessary for urban planning. The analysis of building heights, types, and external components can be helpful in these assessments (Table D11). Street view imagery can also be leveraged for rapid assessment of urban morphology (Table D12).

Table D10 Assessment of Architectural Styles in Mumbai [58] from Street View Images

Style	Period	Description (from street view)
Gothic Revival	19th century	Characterized by pointed arch windows, intricate tracery, and high steeples.
Indo-Saracenic	Late 19th to early 20th century	Features domed roofs, arches, and intricate detailing combining Indian and Gothic elements.
Art Deco	1930s-1940s	Streamlined forms, rounded corners, and horizontal grooves, with facade details incorporating stylized motifs and vibrant colour schemes.
Modern	Post-1947	Minimalist designs with large glass windows and sparse ornamentation.
Contemporary	2000s-present	Innovative materials and designs, often with irregular and dynamic forms.

Table D11 Key Features for Urban Morphology Screening

Feature	Detection from Street View	Relevance
Geometry [19, 26]	Partially	Essential for structural assessment and restoration planning; 2D perspective may limit depth analysis.
Spatial Location [27]	No	Provides context and spatial analysis for comprehensive urban management; can be obtained from metadata.
Building Colour [26]	Yes	Indicative of material condition and environmental effects.
Facade Material [19, 28]	Partially	Crucial for conservation decisions; detectable for exposed materials.
Architectural Style [28, 52, 58]	Partially	Provides insights into the period and cultural context of the building.
Building Height [52, 54, 55]	Yes	Helps to understand vertical and horizontal land utilization, crucial for managing urban expansion and density.
Building Typology [52, 56]	Yes	Identifies the distribution of residential, commercial, and mixed-use areas, influencing urban congestion and land use dynamics.
Visible Condition [52]	Yes	The state of visible infrastructure offers insights into the level of maintenance and investment in densely populated areas.
Commercial Signage/ Advertisements [57]	Yes	Reflects economic activity levels and the commercial density of different city regions.

D.2.5 Challenge 5: Flood Exposure Screening in Rio De Janeiro

Traditional flood vulnerability assessments typically employ data-driven and indicator-based methods [60]. Data-driven approaches analyse historical flood data, property loss records, and building vulnerability to calculate damage rates [61]. This method

Table D12 Urban Morphology-Related Visual Proxies from Street View Images

Feature	Visual Cues	Significance
Number of Storeys	Visible number of floors in a building.	Indicates building height, which can influence urban density and land use.
Residential Buildings	Predominantly residential architecture, often with smaller windows and balconies.	Helps in identifying residential areas, important for urban planning and infrastructure development.
Commercial Buildings	Storefronts, signage, large glass facades.	Identifies commercial zones, which are critical for economic activities and urban zoning.
Mixed-Use Buildings	Combination of storefronts on the lower floors with residential units above.	Highlights areas with mixed land use, essential for understanding urban vitality and pedestrian traffic.
Presence of HVAC Units	Visible external HVAC units, typically on rooftops or building sides.	Indicates active climate control systems, which are significant for energy consumption and building use classification.
Utilities	Presence of utility cables.	Provides insight into the utility services available, infrastructure supporting urban functions, and safety concerns related to overhead wiring.

requires data on flood hazards, building characteristics, and reported losses [62, 63]. In contrast, indicator-based methods create an index system based on the structure and materials of buildings, relying on expert knowledge to assess vulnerability where historical data is scarce [64, 65]. Both methods, while insightful, often depend on costly and time-consuming field surveys. Recent advancements have highlighted the potential of using street view images to enhance flood vulnerability assessments [66–71]. Street view imagery provides information on building characteristics that can support flood exposure screening through facade-derived visual proxies. Important features for assessment include building height, materials, condition, presence of basement windows, number of floors, sill height, roof type, building typology, attachment to adjacent buildings, street slope, occupancy status, number of drains, and lowest floor elevation [60, 67, 72, 73]. Street view imagery serves as an means for identifying a subset of features associated with building flood exposure assessments (Table D13). Integrating street view data with remote sensing techniques can further strengthen flood exposure assessments [60]. While remote sensing provides broad-scale data, street view imagery adds granular detail, such as the condition of building facades that are not visible in aerial imagery. Street view images can also complement hydrological models by providing information on building elevations and facade conditions [67]. In Rio De Janeiro, a city prone to severe flooding due to its topography and climate [74], this visual proxy-based approach is particularly valuable. Traditional methods often miss the finer details of informal settlements and the diverse building types found throughout the city [75] (Table D14). By leveraging street view imagery, urban planners and policymakers can gain a nuanced understanding of the city's spatially explicit flood

exposure visual proxies, enabling more targeted and effective mitigation strategies. For a detailed analysis of the specific visual cues observed for flood exposure, refer to Table D15.

Table D13 Key Features for Flood Exposure Screening.

Feature	Detection from Street View	Relevance
Building Materials [68]	Yes	Different materials react differently to floodwaters, affecting vulnerability.
Building Condition [60, 76]	Yes	Damaged or dilapidated buildings are more vulnerable to flooding.
Presence of Windows at Lower Levels [70]	Yes	Indicates potential for flooding, increasing vulnerability.
Number of Floors [67]	Yes	More floors can provide vertical evacuation options, reducing vulnerability.
Roof Type [77]	Partially	Certain roof types may be more resistant to flood damage.
Building Typology [67]	Yes	Identifies the use of buildings (residential, commercial), influencing flood response strategies.
Attachment to Adjacent Buildings [69]	No	Attached buildings can create barriers or channels for floodwaters.
Street Slope [76]	No	Street slope affects water flow and pooling, influencing flood risk.

Table D14 Assessment of Architectural Styles in Rio De Janeiro [58, 78] from Street View Images

Style	Description (from street view)
Colonial	Characterised by simple, whitewashed facades, often featuring arched windows and doorways, balconies with metal railings, and a symmetrical, understated design.
Neoclassical	Symmetrical facades, arched windows, smooth stucco walls, classical elements like cornices. Note not all buildings may have all features.
Modernist	Emphasis on volume over mass, use of concrete and glass.
Slum/ Informal	Basic construction material, makeshift/ temporary.
Popular	Colourful facades reflecting practical needs with limited decorative elements.

D.2.6 Challenge 6: Heat Exposure and Facade Greening Suitability Screening Cape Town

Traditional methods for assessing heat risk in urban areas often rely on extensive demographic and socioeconomic data gathered through surveys, interviews, and statistical analyses. While comprehensive, these approaches are resource-intensive, time-consuming, and may pose privacy concerns for the communities involved [79]. In response to these limitations, there is increasing interest in using readily available and

Table D15 Flood Exposure Visual Proxies from Street View Images

Feature	Visual Cues	Significance
Building Materials	Type of construction material on facades.	Some materials like brick are more resistant to water damage.
Building Condition	Visible signs of wear or deterioration.	Poor condition indicates higher flood vulnerability.
Presence of Large Openings at Lower Levels	Large windows, doors, or open areas at ground level.	Increases the potential for water ingress during floods.
Number of Floors	Visible floor count.	More floors provide vertical evacuation options.
Water Stains/Peeling Paint/-Mould Presence	Visible signs of water damage on walls.	Indicates past flooding or ongoing moisture issues, increasing vulnerability.
Building Typology	Residential, commercial, or mixed-use.	Helps in planning flood response and evacuation strategies.

more efficient methods, such as street view imagery. This approach provides accessible visual data that can be analysed to infer heat exposure visual proxies. For example, street view images can reveal a building's use type, facade material, construction type, and overall condition, offering qualitative insights into potential heat susceptibility [79] (Table D16). Buildings constructed with certain materials, in poor condition, or lacking specific features may exhibit greater susceptibility to heat, impacting both the structure and its occupants.

Street view imagery can also be used to derive a range of image-based indicators relevant to urban heat exposure, such as the Sky View Factor (SVF) [80], Building View Factor (BVF) [81], Tree View Factor (TVF) [82], and Green View Index (GVI) [81, 82]. These metrics quantify aspects like solar radiation exposure, urban shading, and greenery, which are critical for screening urban heat stress patterns. Street view imagery also offers the potential to identify surfaces suitable for green infrastructure interventions. Blank walls can be assessed for the feasibility of vertical gardens, while flat roofs can be evaluated for the addition of green roofs [79], both of which can help mitigate the severe heat experienced in Cape Town. While these indicators do not replace microclimate simulations or thermal measurements, these visual cues, combined with architectural data (Table D17) and feature identification (Table D18), provide a foundation for more targeted and efficient heat mitigation screening and prioritisation reduction strategies that integrate green facade and roof solutions into the city's evolving architectural landscape.

D.3 Overall Data Flow

Table D19 provides a city-wise summary of data flow through the URBAN-AI pipeline. The table reports the number of candidate street view images retrieved, high-confidence facade instances retained after Grounded-SAM filtering, and the final human-validated GPT-4V outputs used for evaluation.

Table D16 Key Features for Heat Exposure and Facade Greening Suitability Screening.

Feature	Detection from Street View	Relevance
Building Height [83]	Yes	Higher buildings can experience different heat retention levels and shadowing effects.
Building Materials [84, 85]	Yes	Certain materials, such as concrete or metal, can exacerbate heat retention and impact indoor temperatures.
Building Condition [85]	Yes	Poorly maintained buildings may have higher heat infiltration and lower thermal resistance.
Window Type [86]	Yes	Window size, number, and shading can affect indoor temperatures and heat vulnerability.
Roof Type [86]	Partially	Roof characteristics, such as reflective materials or vegetation coverage, can influence heat absorption and cooling potential.
Facade Orientation [85]	Yes	The direction of facades can impact heat gain due to sun exposure throughout the day.
Greenery [85]	No	Surrounding vegetation can mitigate urban heat through shading and cooling effects.

Table D17 Assessment of Architectural Styles in Cape Town [87, 88] from Street View Images

Style	Description (from street view)
Colonial Revival	Symmetry, classical columns, expansive verandas, heavy use of timber and stone.
Victorian	Ornate detailing, steeply pitched roofs, decorative trim, bay windows.
Tudor Revival	Timber framing, steeply pitched gable roofs, decorative half-timbering.
Arts and Crafts	Handcrafted detailing, overhanging eaves.
Modern	Minimalist design, use of glass and steel, flat roofs, functional aesthetic.
Utilitarian	Simple, functional, no specific style. aesthetic.

Appendix E Model Performance and limitations

Despite the effectiveness of URBAN-AI in urban analysis, some limitations highlight areas for improvement. In some cases, the GPT-4V inferred from limited building views, such as when only part of the image was visible, leading to over-generalizations. Removing significantly cropped images can help avoid incomplete assessments.

Identifying materials like PVC, which often mimics wood, proved challenging due to the resolution of the images. Additionally, a significant number of buildings were classified under the broad “other” style category, where plain exteriors and basic construction made it difficult to assign a specific architectural style. Supplementing the model with further contextual information, such as the year of construction, could

Table D18 Heat-Related and Facade Greening Suitability Visual Proxies from Street View Images

Feature	Visual Cues	Significance
Number of floors	Visible floor count.	Taller buildings may experience different heat retention and cooling patterns.
Building Materials	Type of construction material on facades.	Materials like concrete or metal can retain more heat, affecting indoor temperatures.
Building Condition	Visible signs of wear or deterioration.	Poor condition may lead to higher heat infiltration and lower thermal resistance.
Roof Type	Shape of the roof.	Flat roofs can accommodate gardens which can reduce heat absorption and improve cooling.
Blank Walls	Large, unused wall surfaces.	Potential for implementing vertical gardens to reduce heat impact.
Presence of Gutters	Visible gutters and downspouts.	Proper gutter systems can aid in managing rainwater runoff, reducing localized heat build-up through water management and promoting green infrastructure solutions.

Table D19 Data flow and sample sizes across the six study cities.

City	Street View Images	High-Confidence Facade Images	Human-Verified GPT-4V Outputs
Zurich (ZRH)	3,014	1,377	1,345
San Francisco (SF)	8,971	1,242	1,227
Melbourne (MEL)	7,069	1,839	1,786
Mumbai (MUM)	22,872	1,753	1,732
Rio de Janeiro (RJ)	8,065	1,156	1,105
Cape Town (CT)	7,927	1,887	1,861
**Total**	**57,918**	**9,254**	**9,056**

improve the accuracy of these classifications. Mixed-use buildings or structures with hybrid architectural styles posed additional difficulties, as assigning a definitive style was complicated by the mix of design elements. These challenges highlight areas where further training, collaboration with local experts, and improvements in data quality could enhance the model's performance and provide more reliable outputs.

Small architectural features like gutters were inconsistently detected, with some flagged inaccurately or missed altogether, largely due to the limitations of image resolution. In addition, some buildings in good condition were incorrectly labelled as 'poor condition' due to the presence of graffiti. To address this, prompting strategies should be updated to include features like graffiti, which could help refine building condition assessments depending on the project's needs, as these definitions can vary based on the city, context, stakeholder, and project.

The identification of blank wall spaces for vertical gardens in Cape Town was sometimes ambiguous. Implementing clearer definitions, such as a minimum square meter threshold, could enhance accuracy in identifying suitable areas for green infrastructure. Roof type identification was another challenge, particularly when buildings had mixed roof styles (e.g., sloped and flat roofs). The model struggled to handle these variations, suggesting that improved prompting strategies and/or additional data are needed to address such outliers.

Appendix F Supplementary Discussion

- **Chaining Multiple Models:** By chaining pre-trained models, we can achieve high levels of automation without extensive manual labelling, accelerating the urban data collection process while maintaining accuracy. Although full automation through AI reduces the need for traditional labour-intensive tasks, there will still be instances where expert input is required to validate outputs, especially in areas with ambiguous or low-resolution imagery. Nevertheless, chaining these models reduces the overall burden of manual intervention, enabling more scalable and cost-effective urban analysis by allowing different models to address distinct aspects of the data. The cumulative value from combining multiple AI models can drive the development of more comprehensive datasets and provide stakeholders with the ability to generate richer insights, while minimising the need for manual data labelling.
- **Challenges with GPT-4V Output:** The integration of GPT-4V within our URBAN-AI workflow, while instrumental in generating detailed building insights, presents challenges related to the consistency and precision of its outputs. Despite employing structured prompts, we observed that GPT-4V occasionally produces superfluous responses, varying punctuation, or slightly different outputs even with identical prompts. This variability stems from the model's inherent nature to generate diverse language patterns, which can lead to inconsistencies in data interpretation. To address these issues, future efforts should focus on refining prompt engineering techniques, ensuring that prompts are as clear and unambiguous as possible. In addition, fine-tuning parameters such as max_tokens, temperature, and top_p can help improve the specificity and consistency of GPT-4V's responses. The max_tokens parameter limits the length of responses, ensuring concise outputs tailored to the task. Adjusting the temperature to a lower value (closer to 0) reduces randomness, making the outputs more deterministic and focused. Similarly, setting top_p to a lower value prioritises the most probable tokens, refining the diversity of the output while maintaining relevance. Furthermore, implementing post-processing algorithms to standardise outputs can enhance the reliability of the generated data. Continuous iteration and testing of prompt structures will be essential to minimise discrepancies and improve the overall accuracy of GPT-4V in structured data generation tasks.
- **Outdated Repositories:** The quality of information in datasets like Google's Open Buildings varies significantly depending on location, particularly due to issues with imagery completeness. The quality of street view metadata also poses challenges.

The value of the date field in street view panoramas can vary in its granularity, with some records providing the year and month, while others offer only the year. Crowdsourcing efforts, where local communities contribute real-time images of their environments, could fill in gaps in incomplete datasets, particularly in rapidly urbanizing areas where official data collection is lagging.

- **Comparison with Existing Data:** Our analysis revealed that there was no overlap between the heritage status data (gebaeude_gschutzr provided by the City of Zurich) and the high-value historical features identified through AI-driven street view analyses based on architectural style, ornamentation, and materials. This discrepancy may stem from different assessment criteria: municipal heritage designations typically rely on cultural, and legislative considerations, whereas the AI model focuses primarily on visually distinctive attributes. This divergence can uncover gaps in current heritage assessments and identify buildings that may warrant further expert review. Importantly, these outputs should be interpreted as facade-level visual proxies for historical significance rather than formal heritage designations. By identifying visually significant but officially unrecognized buildings, AI can bring attention to overlooked elements. This capability allows for the discovery of architectural gems, unappreciated styles, or lesser-known features that may contribute to a city's heritage narrative. Combining human expertise with AI's analytical capabilities has the potential to create more robust, inclusive, and data-driven approaches to urban planning.
- **Closed vs Open Source Models:** GPT-4V is a closed-source model hosted on OpenAI's servers, which introduces potential privacy concerns. Data, including images and text, must be sent to third-party servers for processing, raising issues around data security and ownership. However, OpenAI is continually updating its privacy policies and offering stricter controls to ensure that users can better manage how their data is handled, mitigating some of these concerns. Despite these challenges, the cost and efficiency of using GPT-4V for urban analysis remain more favourable compared to traditional, labour-intensive manual methods. Additionally, for those seeking greater control over data privacy and lower operational costs, there are continually emerging open-source alternatives such as LLaVA [89] and CogVLM [90]. These models can be downloaded and run on local servers, ensuring data remains entirely in-house and reducing dependency on third-party providers. While these open-source models are smaller and cost-efficient, they are less capable than GPT-4V is today. Despite these trade-offs, both closed and open-source models offer advantages over manual processes, making them viable options depending on the project's specific needs and constraints.
- **Modular Workflow:** As the field of vision and language models rapidly evolves, better models in different parts of the workflow can be switched in to improve performance and accuracy. For instance, newer AI models employing chain-of-thought reasoning [91] can be integrated into the workflow to address current limitations with incomplete or inconsistent data. These models simulate step-by-step logical reasoning, enabling more context-aware analysis and better decision-making even when data is missing or unreliable. This modularity ensures that as AI technology progresses, URBAN-AI remains cutting-edge.

- **Establishing Benchmarks:** Prior to our study, there were no established benchmarks for assessing the performance of vision and language models specifically within the context of construction and urban analysis. Recognising this gap, our research has pioneered the creation of tailored benchmarks that evaluate the efficacy of these models in identifying and classifying building materials, architectural styles, and typologies from facade-level imagery. These benchmarks serve as foundational standards for future researchers, facilitating consistent and comparable assessments across different studies. By establishing these benchmarks, we provide a critical reference point that can drive the development and refinement of AI methodologies in sustainability research.
- **Publishing Ground Truth Data:** A significant contribution of our work is the publication of human-verified facade-level ground truth annotations of building materials, components, and basic architectural data across all six cities studied. This initiative is particularly impactful for cities in the Global South, where such data was previously unavailable or incomplete. By making these datasets publicly accessible, we enable urban planners, policymakers, and researchers to perform more informed and accurate analyses. These datasets enhance the transparency and reproducibility of our study by providing a valuable resource for future research addressing urban sustainability challenges in under-represented regions. The availability of this data supports the creation of a more equitable and inclusive global resource cadastre, fostering urban development worldwide.
- **Human Validation Bias and Class Imbalance:** The evaluation prioritizes human verification to enable assessment across heterogeneous urban contexts where standardized facade-level ground truth data are unavailable. While this allows validation across diverse architectural styles and materials, it introduces subjectivity, particularly for visually ambiguous or minority facade categories such as mixed materials, surface coatings, or partially occluded elements. Lower agreement rates in these cases therefore reflect inherent visual ambiguity and cross-city variability rather than model failure. To mitigate bias, all reviewers followed identical instructions, used a shared visual reference catalogue, and applied a predefined verification rubric. Accordingly, the reported results should be interpreted as qualitative screening accuracy rather than class-balanced predictive performance. Future work may incorporate curated test sets, inter-annotator agreement analysis, or hybrid validation strategies that combine human review with targeted quantitative benchmarks. Similarly, spatial heat maps derived from facade-level indicators may be influenced by uneven street view coverage and should be interpreted as revealing relative spatial patterns and priority areas. Exhaustive and uniformly sampled urban measurements can be pursued in future work as imagery availability improves.
- **Release of URBAN-AI Tool for Immediate Use:** The release of the URBAN-AI tool allows stakeholders to use the methodology in real-time urban planning and sustainability initiatives. Stakeholders can customize prompts and incorporate diverse datasets, including interior imagery or high-resolution drone-captured images, to enhance the tool's applicability across various contexts. This flexibility ensures that URBAN-AI can adapt to different urban environments and specific project requirements, making it a versatile tool for a wide range of applications. By

enabling users to modify prompts and integrate additional data sources, URBAN-AI empowers stakeholders to conduct more nuanced and context-specific analyses, facilitating more effective urban management practices.

- **Overlaying External Datasets:** Integrating external datasets with URBAN-AI can significantly enhance the depth and breadth of urban analysis. For example, overlaying topographical data in flood vulnerable areas of Rio de Janeiro can provide a more comprehensive understanding of how terrain influences flood risks. Similarly, incorporating resident data in urban morphology studies or seismic zone information in seismic retrofit potential assessments can refine our analyses, offering more targeted and actionable insights. By combining multiple data layers, URBAN-AI can deliver a more holistic view of urban environments, with facade-derived indicators acting as an upstream screening layer that complements, rather than replaces, domain-specific models.
- **Granularity of Analysis:** While Grounding-SAM effectively segments building facades, it is less adept at identifying smaller architectural features such as doors, windows, and balconies. To achieve higher precision, we plan to explore advanced vision models like Meta's Segment Anything [92], which offers more detailed segmentation capabilities. Additionally, clustering the collected data could lead to the generation of architectural archetypes, providing deeper insights into urban layouts and material usage patterns. These advancements will enable more precise urban-level screening of energy assessments and support sustainable urban management by offering a nuanced understanding of material properties and architectural elements. While URBAN-AI proves valuable for high-level decision-making using street view imagery, on-site audits and thorough human-led inspections remain essential for obtaining a greater level of detail. Therefore, integrated approaches, combining AI-driven analysis with on-site inspections can lead to more accurate and effective sustainability strategies.
- **Policy and Industry Implications:** The observed disparities in data availability, image coverage, and metadata quality across cities highlight the need for coordinated policy and industry action to support open, regularly updated urban data infrastructures, particularly in low- and middle-income countries. URBAN-AI demonstrates how facade-level visual proxies can partially bridge these gaps, but long-term impact requires complementary investments in open data standards, improved street-level imagery coverage, and capacity building for local authorities. These implications reinforce the role of AI-based screening tools as enabling layers within broader urban governance and sustainability frameworks, rather than as standalone decision-making systems.
- **Future Applications:** In the Global North, URBAN-AI can be used to match supply and demand for reusable construction materials in a structured and efficient manner. By analysing street view imagery and urban building data, URBAN-AI can generate inventories indicating the presence of materials available from demolition sites, renovations, and construction projects. These inventories can identify the types of materials present as visual indications of potential availability, but may require further detailed manual audits for obtaining the exact quantities of the materials. The information can be cross-referenced with ongoing construction

or retrofitting needs, facilitating organised exchanges between material suppliers and users, recycling centres, and other stakeholders. In the Global South, where formal material exchanges and structured systems are less prevalent, URBAN-AI can be applied in a more informal yet impactful way through the development of a waste picker cadastre. The AI-driven insights from imagery can provide indicative, location-aware visual information, helping waste pickers locate and recover materials that would otherwise be discarded. This bottom-up approach not only promotes circular construction practices but also supports the livelihoods of waste pickers, fostering a more equitable and inclusive circular economy in regions where formal systems for resource management are lacking.

References

[1] World Bank: World Bank Open Data. https://data.worldbank.org (2015)

[2] United Nations Framework Convention on Climate Change (UNFCCC): Paris Agreement. Adopted at COP21, Paris, Bonn, Germany (2015)

[3] Black, S., Parry, I.W.H., Zhunussova, K.: Is the Paris Agreement Working? A Stocktake of Global Climate Mitigation. Imf working paper, International Monetary Fund, Washington, DC (2023)

[4] Raghu, D., De Wolf, C.: India's Informal Reuse Ecosystem Towards Circular Construction. In: Design for Rethinking Resources, pp. 127–137. Springer, Cham, Switzerland (2023). https://doi.org/10.1007/978-3-031-36554-6_10

[5] Honic, M., Ferschin, P., Breitfuss, D., Cencic, O., Gourlis, G., Kovacic, I., De Wolf, C.: Framework for the assessment of the existing building stock through bim and gis. Developments in the Built Environment **13**, 100110 (2023)

[6] Minunno, R., O'Grady, T., Morrison, G.M., Gruner, R.L.: Exploring environmental benefits of reuse and recycle practices: A circular economy case study of a modular building. Resources, Conservation and Recycling **160**, 104855 (2020)

[7] Heisel, F., Rau-Oberhuber, S.: Calculation and evaluation of circularity indicators for the built environment using the case studies of umar and madaster. Journal of Cleaner Production **243**, 118482 (2020)

[8] Schneider, M., Rubli, S., Gugerli, H., Bürgi, M.: A model for resource management of mineral materials in the city of zurich (2007)

[9] Hashimoto, S., Tanikawa, H., Moriguchi, Y.: Framework for estimating potential wastes and secondary resources accumulated within an economy–a case study of construction minerals in japan. Waste Management **29**(11), 2859–2866 (2009)

[10] Wiedenhofer, D., Steinberger, J.K., Eisenmenger, N., Haas, W.: Maintenance and

expansion: modeling material stocks and flows for residential buildings and transportation networks in the eu25. Journal of Industrial Ecology **19**(4), 538–551 (2015)

[11] Meinel, G., Hecht, R., Herold, H.: Analyzing building stock using topographic maps and gis. Building Research & Information **37**(5-6), 468–482 (2009)

[12] Kleemann, F., Lederer, J., Rechberger, H., Fellner, J.: Gis-based analysis of vienna's material stock in buildings. Journal of Industrial Ecology **21**(2), 368–380 (2017)

[13] Ortlepp, R., Gruhler, K., Schiller, G.: Materials in germany's domestic building stock: calculation model and uncertainties. Building Research & Information **46**(2), 164–178 (2018)

[14] Mohammadiziazi, R., Bilec, M.M.: Quantifying and spatializing building material stock and renovation flow for circular economy. Journal of Cleaner Production **389**, 135765 (2023)

[15] Chen, J., Lu, W., Xue, F.: Looking beneath the surface: A visual-physical feature hybrid approach for unattended gauging of construction waste composition. Journal of environmental management **286**, 112233 (2021)

[16] Ku, Y., Yang, J., Fang, H., Xiao, W., Zhuang, J.: Deep learning of grasping detection for a robot used in sorting construction and demolition waste. Journal of Material Cycles and Waste Management **23**, 84–95 (2021)

[17] Sun, Y., Gu, Z.: Using computer vision to recognize construction material: A trustworthy dataset perspective. Resources, Conservation and Recycling **183**, 106362 (2022)

[18] Raghu, D., Bucher, M.J.J., De Wolf, C.: Towards a 'resource cadastre' for a circular economy–urban-scale building material detection using street view imagery and computer vision. Resources, Conservation and Recycling **198**, 107140 (2023)

[19] Del Bo, A., Bignami, D.F.: Sustainable Social, Economic and Environmental Revitalization in Multan City. Research for Development. Springer, Cham, Switzerland (2014). https://doi.org/10.1007/978-3-319-02117-1

[20] Liu, S., Bin Mamat, M.J.: Application of 3d laser scanning technology for mapping and accuracy assessment of the point cloud model for the great achievement palace heritage building. Heritage Science **12**(1), 153 (2024)

[21] Kıvılcım, C., Duran, Z.: A semi-automated point cloud processing methodology for 3d cultural heritage documentation. The International Archives of the Photogrammetry, Remote Sensing and Spatial Information Sciences **41**, 293–296 (2016)

[22] Angeloni, R., Mariotti, C., Petetta, L., Coppetta, L., *et al.*: Enabling scan-to-bim workflow for heritage conservation and management process. The International Archives of the Photogrammetry, Remote Sensing and Spatial Information Sciences **48**, 79–86 (2023)

[23] Fan, Z., Zhang, F., Loo, B.P., Ratti, C.: Urban visual intelligence: Uncovering hidden city profiles with street view images. Proceedings of the National Academy of Sciences **120**(27), 2220417120 (2023)

[24] Wang, Y., Wang, S., Pan, Y., Li, C., Chen, C., Wang, J.: Immersive virtual reality and computer vision for heritage: visual evaluation and perception of the industrial heritage sites along the yunnan–vietnam railway (yunnan section). Heritage Science **12**(1), 36 (2024)

[25] Kurz, D.: Die Disziplinierung der Stadt: Moderner Städtebau in Zürich 1900 Bis 1940. gta Verlag, Zürich (2022)

[26] De Fino, M., Galantucci, R.A., Fatiguso, F.: Condition assessment of heritage buildings via photogrammetry: A scoping review from the perspective of decision makers. Heritage **6**(11), 7031–7066 (2023)

[27] García-Esparza, J.A., Tena, P.A.: A gis-based methodology for the appraisal of historical, architectural, and social values in historic urban cores. Frontiers of Architectural Research **9**(4), 900–913 (2020)

[28] Khattak, N., Derakhshan, H., Thambiratnam, D.P., Perera, N.J., Ingham, J.M.: Using heritage building registers to characterise unreinforced masonry buildings of brisbane, australia. Australian Journal of Structural Engineering **24**(1), 1–23 (2023)

[29] Upper Lough Erne: Heritage Asset Audit. Technical report, Upper Lough Erne Landscape Partnership, Northern Ireland

[30] Ching, F.D.K.: Architecture: Form, Space, and Order, 5th edn. John Wiley & Sons, Hoboken, NJ (2023)

[31] Nicodemo, G., Pittore, M., Masi, A., Manfredi, V.: Modelling exposure and vulnerability from post-earthquake survey data with risk-oriented taxonomies: Aedes form, gem taxonomy and ems-98 typologies. International Journal of Disaster Risk Reduction **50**, 101894 (2020)

[32] Federal Emergency Management Agency (FEMA): Rapid Visual Screening of Buildings for Potential Seismic Hazards: A Handbook. Technical report, U.S. Department of Homeland Security, Washington, DC (2017)

[33] Wang, C., Antos, S.E., Triveno, L.M.: Automatic detection of unreinforced masonry buildings from street view images using deep learning-based image

segmentation. Automation in Construction **132**, 103968 (2021)

[34] Kalfarisi, R., Hmosze, M., Wu, Z.Y.: Detecting and geolocating city-scale soft-story buildings by deep machine learning for urban seismic resilience. Natural Hazards Review **23**(1), 04021062 (2022)

[35] Wang, C., Hornauer, S., Yu, S.X., McKenna, F., Law, K.H.: Instance segmentation of soft-story buildings from street-view images with semiautomatic annotation. Earthquake Engineering & Structural Dynamics **52**(8), 2520–2532 (2023)

[36] Field, E.H., et al.: UCERF3: A New Earthquake Forecast for California's Complex Fault System. Technical report, U.S. Geological Survey, Reston, VA (2015)

[37] Federal Emergency Management Agency (FEMA): National Incident Management System. U.S. Department of Homeland Security, Washington, DC (2017)

[38] San Francisco Planning Department: Preservation Bulletin: Architectural Periods and Styles in San Francisco. http://www.sfplanning.org. Accessed: 12-07-2024 (2024)

[39] Ghione, F., Mæland, S., Meslem, A., Oye, V.: Building stock classification using machine learning: A case study for oslo, norway. Frontiers in Earth Science **10**, 886145 (2022)

[40] Nascimbene, R.: Investigation of seismic damage to existing buildings by using remotely observed images. Engineering Failure Analysis **161**, 108282 (2024)

[41] Moniz, N., Vale, Z., Cascalho, J., Silva, C., Sebastião, R. (eds.): Progress in Artificial Intelligence: 22nd EPIA Conference on Artificial Intelligence, EPIA 2023, Faial Island, Azores, September 5–8, 2023, Proceedings, Part II. Lecture Notes in Computer Science, vol. 14116. Springer, Cham, Switzerland (2024)

[42] Abela, A., Hoxley, M., McGrath, P., Goodhew, S.: An investigation of the appropriateness of current methodologies for energy certification of mediterranean housing. Energy and Buildings **130**, 210–218 (2016)

[43] Seyedzadeh, S., Rahimian, F.P., Glesk, I., Roper, M.: Machine learning for estimation of building energy consumption and performance: a review. Visualization in Engineering **6**, 1–20 (2018)

[44] Xu, F., Wong, M.S., Zhu, R., Heo, J., Shi, G.: Semantic segmentation of urban building surface materials using multi-scale contextual attention network. ISPRS Journal of Photogrammetry and Remote Sensing **202**, 158–168 (2023)

[45] Orenga Panizza, R., Nik-Bakht, M.: Extraction of energy-influential parameters from building façade images through google street view. In: EC3 Conference 2023,

vol. 4, pp. 0–0 (2023). European Council on Computing in Construction

[46] Lan, H., Gou, Z., Xie, X.: A simplified evaluation method of rooftop solar energy potential based on image semantic segmentation of urban streetscapes. Solar Energy **230**, 912–924 (2021)

[47] City of Melbourne: Building towards zero carbon target. https://www.melbourne.vic.gov.au/news-and-media/Pages/Building-towards-zero-carbon-target.aspx. Accessed: 2024-07-20 (2024)

[48] Jellis Craig: The Many Facades of Melbourne. https://www.jelliscraig.com.au/blog/the-many-facades-of-melbourne. Accessed: 2024-07-20 (2024)

[49] Mastercraft Construction: Period & Heritage Home Architecture in Melbourne. https://www.mastercraftconstruction.com.au/period-heritage-home-architecture-melbourne/. Accessed: 2024-07-20 (2024)

[50] Sun, M., Bardhan, R.: Identifying hard-to-decarbonize houses from multi-source data in cambridge, uk. Sustainable Cities and Society **100**, 105015 (2024)

[51] Sun, M., Han, C., Nie, Q., Xu, J., Zhang, F., Zhao, Q.: Understanding building energy efficiency with administrative and emerging urban big data by deep learning in glasgow. Energy and buildings **273**, 112331 (2022)

[52] Biljecki, F., Ito, K.: Street view imagery in urban analytics and gis: A review. Landscape and Urban Planning **215**, 104217 (2021)

[53] Kraff, N.J., Wurm, M., Taubenböck, H.: The dynamics of poor urban areas-analyzing morphologic transformations across the globe using earth observation data. Cities **107**, 102905 (2020)

[54] Gao, Y., Shahab, S., Ahmadpoor, N.: Morphology of urban villages in china: A case study of dayuan village in guangzhou. Urban Science **4**(2), 23 (2020)

[55] Marinovic, G.I.: Urban morphology of informally built dwellings: counter-mapping coastal cities of montenegro. Archnet-IJAR: International Journal of Architectural Research **17**(4), 665–685 (2023)

[56] Kang, J., Körner, M., Wang, Y., Taubenböck, H., Zhu, X.X.: Building instance classification using street view images. ISPRS journal of photogrammetry and remote sensing **145**, 44–59 (2018)

[57] Sharifi Noorian, S., Qiu, S., Psyllidis, A., Bozzon, A., Houben, G.-J.: Detecting, classifying, and mapping retail storefronts using street-level imagery. In: Proceedings of the 2020 International Conference on Multimedia Retrieval, pp. 495–501 (2020)

[58] Art Deco Mumbai: Exploring Art Deco Architecture in Mumbai. Accessed: 2024-07-10 (2024). https://www.artdecomumbai.com/

[59] Art Deco Mumbai: Bombay's Transition to Modernity: The Dawn of Art Deco in Bombay. Accessed: 2024-07-10 (2024). https://www.artdecomumbai.com/research/bombays-transition-to-modernity-the-dawn-of-art-deco-in-bombay/

[60] Xing, Z., Yang, S., Zan, X., Dong, X., Yao, Y., Liu, Z., Zhang, X.: Flood vulnerability assessment of urban buildings based on integrating high-resolution remote sensing and street view images. Sustainable Cities and Society **92**, 104467 (2023)

[61] Papathoma-Köhle, M., Schlögl, M., Dosser, L., Roesch, F., Borga, M., Erlicher, M., Keiler, M., Fuchs, S.: Physical vulnerability to dynamic flooding: Vulnerability curves and vulnerability indices. Journal of Hydrology **607**, 127501 (2022)

[62] Amadio, M., Scorzini, A.R., Carisi, F., Essenfelder, A.H., Domeneghetti, A., Mysiak, J., Castellarin, A.: Testing empirical and synthetic flood damage models: the case of italy. Natural Hazards and Earth System Sciences **19**(3), 661–678 (2019)

[63] Pham, Q.B., Ali, S.A., Bielecka, E., Calka, B., Orych, A., Parvin, F., Łupikasza, E.: Flood vulnerability and buildings' flood exposure assessment in a densely urbanised city: comparative analysis of three scenarios using a neural network approach. Natural Hazards **113**(2), 1043–1081 (2022)

[64] Ciurean, R., Hussin, H., Van Westen, C., Jaboyedoff, M., Nicolet, P., Chen, L., Frigerio, S., Glade, T.: Multi-scale debris flow vulnerability assessment and direct loss estimation of buildings in the eastern italian alps. Natural hazards **85**, 929–957 (2017)

[65] Park, K., Choi, S.-H., Yu, I.: Risk type analysis of building on urban flood damage. Water **13**(18), 2505 (2021)

[66] Sorboni, N.G., Wang, J., Najafi, M.R.: Automated first floor height estimation for flood vulnerability analysis using deep learning and google street view. Journal of Flood Risk Management **17**(2), 12975 (2024)

[67] Velez, R., Calderon, D., Carey, L., Aime, C., Hultquist, C., Yetman, G., Kruczkiewicz, A., Gorokhovich, Y., Chen, R.S.: Advancing data for street-level flood vulnerability: Evaluation of variables extracted from google street view in quito, ecuador. IEEE Open Journal of the Computer Society **3**, 51–61 (2022)

[68] Diakakis, M., Deligiannakis, G., Pallikarakis, A., Skordoulis, M.: Identifying elements that affect the probability of buildings to suffer flooding in urban areas using google street view. a case study from athens metropolitan area in greece. International journal of disaster risk reduction **22**, 1–9 (2017)

[69] Blanco-Vogt, A., Schanze, J.: Assessment of the physical flood susceptibility of buildings on a large scale–conceptual and methodological frameworks. Natural Hazards and Earth System Sciences **14**(8), 2105–2117 (2014)

[70] Müller, A., Reiter, J., Weiland, U.: Assessment of urban vulnerability towards floods using an indicator-based approach–a case study for santiago de chile. Natural Hazards and Earth System Sciences **11**(8), 2107–2123 (2011)

[71] Mollaei, A., Ibrahim, N., Habib, K.: Estimating the construction material stocks in two canadian cities: A case study of kitchener and waterloo. Journal of Cleaner Production **280**, 124501 (2021)

[72] Ning, H., Li, Z., Ye, X., Wang, S., Wang, W., Huang, X.: Exploring the vertical dimension of street view image based on deep learning: a case study on lowest floor elevation estimation. International Journal of Geographical Information Science **36**(7), 1317–1342 (2022)

[73] Aahlaad, M., Mozumder, C., Tripathi, N., Pal, I.: An object-based image analysis of worldview-3 image for urban flood vulnerability assessment and dissemination through esri story maps. Journal of the Indian Society of Remote Sensing **49**(11), 2639–2654 (2021)

[74] Altafini, D., Costa Braga, A., Ugalde, C.: Mapping urban flood-prone areas' spatial structure and their tendencies of change: A network study for brazil's porto alegre metropolitan region. Cartographica: The International Journal for Geographic Information and Geovisualization **58**(4), 205–226 (2023)

[75] Architecture History: Rio de Janeiro, Brazil. Accessed: 2024-11-12 (n.d.). http://architecture-history.org/schools/RIO%20DE%20JANEIRO,%20BRAZIL.html

[76] Fedeski, M., Gwilliam, J.: Urban sustainability in the presence of flood and geological hazards: The development of a gis-based vulnerability and risk assessment methodology. Landscape and urban planning **83**(1), 50–61 (2007)

[77] Angela, B.-V., Norbert, H., Jochen, S.: Building extraction from remote sensing data for parameterising a building typology: a contribution to flood vulnerability assessment. In: Joint Urban Remote Sensing Event 2013, pp. 147–150 (2013). IEEE

[78] Re-Thinking The Future: Past, Present, and Future: Architecture of Brazil. Accessed: 2024-11-12 (n.d.). https://www.re-thinkingthefuture.com/city-and-architecture/a5495-past-present-and-future-architecture-of-brazil/

[79] Wang, C., Antos, S.E., Gosling-Goldsmith, J.G., Triveno, L.M., Zhu, C., Meding, J., Ye, X.: Assessing climate disaster vulnerability in peru and colombia using street view imagery: A pilot study. Buildings **14**(1), 14 (2023)

[80] Chiang, Y.-C., Liu, H.-H., Li, D., Ho, L.-C.: Quantification through deep learning of sky view factor and greenery on urban streets during hot and cool seasons. Landscape and Urban Planning **232**, 104679 (2023)

[81] Chen, K., Tian, M., Zhang, J., Xu, X., Yuan, L.: Evaluating the seasonal effects of building form and street view indicators on street-level land surface temperature using random forest regression. Building and Environment **245**, 110884 (2023)

[82] Hu, Y., Qian, F., Yan, H., Middel, A., Wu, R., Zhu, M., Han, Q., Zhao, K., Wang, H., Shao, F., *et al.*: Which street is hotter? street morphology may hold clues-thermal environment mapping based on street view imagery. Building and Environment **262**, 111838 (2024)

[83] Zou, Q., Yang, J., Zhang, Y., Bai, Y., Wang, J.: Variation in community heat vulnerability for shenyang city under local climate zone perspective. Building and Environment, 112242 (2024)

[84] Tabatabaei, S.S., Fayaz, R.: The effect of facade materials and coatings on urban heat island mitigation and outdoor thermal comfort in hot semi-arid climate. Building and Environment **243**, 110701 (2023)

[85] Samuelson, H., Baniassadi, A., Lin, A., González, P.I., Brawley, T., Narula, T.: Housing as a critical determinant of heat vulnerability and health. Science of the total environment **720**, 137296 (2020)

[86] Loga, T., Stein, B., Diefenbach, N.: Tabula building typologies in 20 european countries—making energy-related features of residential building stocks comparable. Energy and Buildings **132**, 4–12 (2016)

[87] Coetzer, N.R.: The Production of the City as a White Space—representing and Restructuring Identity and Architecture, Cape Town, 1892–1936. University of London, University College London (United Kingdom), ??? (2004)

[88] Van Graan, A.: Negotiating modernism in cape town: 1918-1948: an investigation into the introduction, contestation, negotiation and adaptation of modernism in the architecture of cape town (2011)

[89] Liu, H., Li, C., Wu, Q., Lee, Y.J.: Visual instruction tuning. Advances in neural information processing systems **36** (2024)

[90] Wang, W., Lv, Q., Yu, W., Hong, W., Qi, J., Wang, Y., Ji, J., Yang, Z., Zhao, L., Song, X., et al.: Cogvlm: Visual expert for pretrained language models. arXiv preprint arXiv:2311.03079 (2023)

[91] OpenAI: Learning to Reason with Large Language Models. Accessed: 2024-10-09 (2024). https://openai.com/index/learning-to-reason-with-llms/

[92] Kirillov, A., Mintun, E., Ravi, N., Mao, H., Rolland, C., Gustafson, L., Xiao, T., Whitehead, S., Berg, A.C., Lo, W.-Y., *et al.*: Segment anything. In: Proceedings of the IEEE/CVF International Conference on Computer Vision, pp. 4015–4026 (2023)
